# A Computational Model of Inferior Colliculus Responses to Amplitude Modulated Sounds in Young and Aged Rats

**DOI:** 10.3389/fncir.2012.00077

**Published:** 2012-11-02

**Authors:** Cal F. Rabang, Aravindakshan Parthasarathy, Yamini Venkataraman, Zachery L. Fisher, Stephanie M. Gardner, Edward L. Bartlett

**Affiliations:** ^1^Weldon School of Biomedical Engineering, Purdue UniversityWest Lafayette, IN, USA; ^2^Department of Biological Sciences, Purdue UniversityWest Lafayette, IN, USA

**Keywords:** aging, neuron, inhibition, amplitude modulation, auditory, GABA, lateral lemniscus, superior olive

## Abstract

The inferior colliculus (IC) receives ascending excitatory and inhibitory inputs from multiple sources, but how these auditory inputs converge to generate IC spike patterns is poorly understood. Simulating patterns of *in vivo* spike train data from cellular and synaptic models creates a powerful framework to identify factors that contribute to changes in IC responses, such as those resulting in age-related loss of temporal processing. A conductance-based single neuron IC model was constructed, and its responses were compared to those observed during *in vivo* IC recordings in rats. IC spike patterns were evoked using amplitude-modulated tone or noise carriers at 20–40 dB above threshold and were classified as low-pass, band-pass, band-reject, all-pass, or complex based on their rate modulation transfer function tuning shape. Their temporal modulation transfer functions were also measured. These spike patterns provided experimental measures of rate, vector strength, and firing pattern for comparison with model outputs. Patterns of excitatory and inhibitory synaptic convergence to IC neurons were based on anatomical studies and generalized input tuning for modulation frequency. Responses of modeled ascending inputs were derived from experimental data from previous studies. Adapting and sustained IC intrinsic models were created, with adaptation created via calcium-activated potassium currents. Short-term synaptic plasticity was incorporated into the model in the form of synaptic depression, which was shown to have a substantial effect on the magnitude and time course of the IC response. The most commonly observed IC response sub-types were recreated and enabled dissociation of inherited response properties from those that were generated in IC. Furthermore, the model was used to make predictions about the consequences of reduction in inhibition for age-related loss of temporal processing due to a reduction in GABA seen anatomically with age.

## Introduction

The inferior colliculus (IC) is a major integrative center of auditory processing, receiving multiple ascending excitatory inputs from the contralateral ventral and dorsal cochlear nuclei (VCN and DCN, respectively) and from the contralateral lateral and medial superior olive (LSO and MSO) to form different functional zones within the IC (reviewed in Kelly and Caspary, 2005). These excitatory input patterns are superimposed upon similar functional zones formed by the inhibitory dorsal and ventral nuclei of the lateral lemniscus (DNLL and VNLL), as well as the superior olivary nucleus (Cant and Benson, 2006; Loftus et al., 2010). In addition, there are excitatory inputs from the contralateral IC, a network of inhibitory interneurons within the IC, local excitatory collaterals, and descending projections from auditory cortex (Oliver et al., 1991; Paloff et al., 1992; Coomes Peterson and Schofield, 2007). Excitatory projections are glutamatergic, acting on AMPA and NMDA receptors in the IC neurons (Adams and Wenthold, 1979; Wu et al., 2004). Inhibitory projections are primarily GABAergic, with a few glycinergic inputs (Caspary et al., 1990b; Winer et al., 1995; Helfert et al., 1999). This complex interplay of excitatory and inhibitory projections from various nuclei of the auditory pathway makes the IC an important nucleus for understanding how neural representations are transformed as they ascend the auditory pathway.

Age-related central auditory changes in auditory processing primarily manifest as deficits in temporal processing. This has been observed psychophysically in humans, where older listeners perform significantly worse than younger listeners in gap detection tasks (Schneider et al., 1994) or in word recognition tasks (Frisina and Frisina, 1997; Strouse et al., 1998) even when they have comparable hearing thresholds. These deficits are exacerbated in the presence of competing sounds or in the presence of background noise (Frisina and Frisina, 1997). We have previously demonstrated age-related deficits in temporal processing using envelope following responses in Fischer-344 rats. The strength of phase locking to the amplitude modulation showed age-related deficits primarily for faster modulation frequencies (Parthasarathy and Bartlett, 2012), under reduced modulation depth (Parthasarathy and Bartlett, 2011) or in the presence of background noise (Parthasarathy et al., 2010). At the level of the single neuron, this has been observed in the cochlear nuclei (Schatteman et al., 2008) as well as the IC. One study in IC showed that aged animals had fewer units with rate coding for higher modulation frequencies compared to young animals, which had rate best modulation frequencies (rBMFs) >100 Hz and an increase in overall spike counts at low modulation frequencies (Walton et al., 2002). Other studies have also shown a shift in rate coding from band-pass to low-pass in aged animals (Palombi et al., 2001), as well as a increased in minimum gap thresholds for gap detection stimuli (Walton et al., 1998).

Inferior colliculus neurons represent sinusoidal amplitude-modulated tones (sAM) or noise (nAM) with action potentials synchronized to the stimulus modulation envelope. The IC is the first nucleus in the auditory pathway where rate coding becomes prominent and prevalent, with many neurons exhibiting band-pass (BP) coding (firing rate maximum tuned to a modulation frequency), band-reject (BR) coding (firing rate minimum tuned to a modulation frequency) or low-pass coding in discharge rate in addition to their temporally synchronized responses (Rees and Moller, 1987; Langner and Schreiner, 1988; Krishna and Semple, 2000). The neural mechanisms by which rate tuning for AM frequency emerge in IC remain unresolved. One approach to understanding the basis for IC responses is through computational models that incorporate synaptic and intrinsic properties measured *in vitro* to investigate how these mechanisms help generate auditory responses recorded *in vivo*. Previous IC models adjusted existing neuron models of cochlear nuclei to match physiological characteristics (Cai et al., 1998a,b) or were phenomenological models intended to explain experimental observations (Hewitt and Meddis, 1994; Nelson and Carney, 2004; Guerin et al., 2006). Although they did provide insight into potential input schemes for generating rate coded IC neurons, these models did not take into account IC-specific cellular properties, synaptic mechanisms, or short-term synaptic plasticity. A computational model of the IC which incorporates these elements and which can be validated against neuronal responses from the IC would help provide a better understanding of the neural mechanisms of temporal processing in the IC. This model can then be used to predict synaptic mechanisms that can replicate altered neural encoding of sound stimuli in aging.

In this study we have created single compartment models of IC neurons that use input trains derived from data recorded from LSO, DNLL, VNLL, DCN, and VCN from previous studies. We have also recorded single unit responses of IC neurons to AM stimuli from young and aged animals. Together, the input, synaptic and intrinsic properties of the models were able to reproduce the most common response types of the IC to these AM stimuli. Using these IC neuron models as a template, we have then attempted to change these input and synaptic properties, especially those pertaining to synaptic inhibition, to simulate the responses of the aged animals as observed in our study as well as from previous other studies.

## Materials and Methods

### Single unit recordings

#### Surgical procedures

Five young (4–6 months) and 4 aged (22–24 months) Fischer-344 rats were used in this study. All surgical protocols used were approved by the Purdue University animal care and use committee (PACUC 06-106). Auditory brainstem responses and frequency following responses were recorded from these animals a few days prior to surgery, to ensure all animals had hearing thresholds typical for their age and there were no signs of any other abnormal auditory pathologies. Surgeries and recordings were performed in a 9′ × 9′ double walled acoustic chamber (Industrial Acoustics Corporation). Anesthesia was induced in the animals using a mixture of ketamine (VetaKet, 80 mg/kg for the young, 60 mg/kg for the aged) and medetomidine (Dexdomitor, 0.2 mg/kg for the young, and 0.1 mg/kg for the aged) administered intra-muscularly via injection. The reduced concentration of anesthesia for the aged was to account for their decreased liver function. The animals were maintained on oxygen through a manifold. The pulse rate and oxygen saturation were maintained using a pulse-oximeter to ensure they were within normal ranges. Supplementary doses of the anesthesia (20 mg/kg of ketamine, 0.05 mg/kg of medetomidine) were administered intra-muscularly as required to maintain areflexia and a surgical plane of anesthesia. An initial dose of dexamethasone and atropine was administered to reduce swelling and mucosal secretions. A constant physiological body temperature was maintained using a water-circulated heating pad (Gaymar) set at 37°C with the pump placed outside the recording chamber to eliminate audio and electrical interferences. A central incision was made along the midline, and the calvaria exposed. A stainless steel headpost was secured anterior to bregma using three screws drilled into the skull and a head-cap constructed of orthodontic resin (Dentsply). A craniotomy was performed posterior to the lambda suture and 1 mm lateral from the midline. The dura mater was kept intact, and the site of recording was estimated stereotaxically using a rat atlas (Paxinos and Watson, 2006) as well as using internal vasculature landmarks and physiological measurements.

#### Stimulus description and recording procedures

Sound stimuli were generated using SigGenRP (Tucker-Davis Technologies, TDT) at a 97.64 kHz sampling rate (standard TDT sampling rate) and presented through custom-written interfaces in OpenEx software (TDT) in a random order for each repetition. Sound waveforms were generated via a multichannel processor (RX6, TDT), amplified (SA1, TDT), and presented free-field through a Bowers and Wilkins DM601 speaker. The sounds were presented to the animal at azimuth 0° and elevation 0°, calibrated at a distance of 115 cm from speaker to ear, using a Bruel and Kjaer microphone and SigCal software (TDT). All stimuli used had a 5 ms cosine squared gate at onset and offset. Search stimuli used were 200 ms long BP filtered noise with center frequencies from 1 to 36 kHz in five steps per octave with a 0.5 octave bandwidth. The stimuli for the tuning curve were 200 ms long pure tones with frequencies from 500 to 40 kHz, with 10 steps per octave. Filtered noise and tuning curve stimuli were presented every 800 ms. The rate-level stimuli consisted of 100 or 200 ms long pure tones set at the center frequency (CF) of the neuron presented at varying sound levels from 5 to 85 dB SPL in 10 dB steps. Sinusoidally amplitude-modulated noise (nAM) and tone (sAM) stimuli were 750 ms long, with modulation frequency ranging from 8 to 1024 Hz in one octave steps. The nAM stimuli used broadband Gaussian noise as the carrier (0.1–44 kHz), while the carrier frequency of the sAM stimuli were set to the CF of the neuron that was isolated. AM stimuli were presented every 2000–2500 ms and were 100% modulated.

Single unit activity and multiunit activity in the IC were recorded using a tungsten electrode (A-M Systems) encased in a glass capillary that was advanced using a hydraulic micro-drive (Narishige). The IC was identified based on short-latency driven responses to the 1/2 octave band-passed noise search stimuli. The central nucleus of the IC was identified using the ascending tonotopy, as well as narrowly tuned responses to pure tones of various frequencies. Once an auditory neuron was isolated using the search stimuli, a tuning curve was obtained to determine the CF of the neuron. Responses of the neuron to 5–10 repetitions of each sound stimulus were recorded (usually five repetitions for the tuning curve and 10 for other stimuli). Once the CF was determined, the responses of the neuron were obtained to nAM stimuli or sAM stimuli with the carrier frequency set at CF. The sound level of presentation for the sAM and nAM stimuli were set at the lowest sound level that produced a robust sustained response to the tone set at CF. This usually was 20–40 dB above threshold and corresponded to about 60–70 dB SPL for the young and 75–85 dB for the aged, comparable to the sound levels used in our previous studies of the envelope following responses in young and aged animals (Parthasarathy et al., 2010; Parthasarathy and Bartlett, 2011, 2012).

#### Stimulus generation, data acquisition, and recording

Neural signals were acquired using the tungsten electrode connected to a headstage (RA4, TDT) and amplified (RA4PA preamplifier, TDT). The digitized waveforms and spike times were recorded with a multichannel recording and stimulation system (RZ-5, TDT) at a sampling rate of 24.41 kHz (standard TDT sampling rate). The interface for acquisition and spike sorting were custom made using the OpenEx and RPvdsEx software (TDT). The single units acquired were filtered between 300 and 5000 Hz. Single units that were substantially above noise threshold were sorted visually online and then subsequently identified and isolated offline using the OpenEx interface based on waveform similarity. Typically the isolated single units had a signal-to-noise ratio of at least 6 dB. The acquired spikes were stored in data tank and analyzed using custom-written software in MATLAB. Offline sorting of the spike waveforms was performed in OpenExplorer (TDT) if necessary to isolate consistent single units from random background activity.

#### Data analysis and response type classification

Spontaneous rate was calculated as the mean rate of the 200 ms period that preceded each trial of the stimulus presentation. An auditory driven neuron was defined as a neuron that exhibited an overall firing rate that was at least two standard deviations (SD) higher than the spontaneous firing rate during the presentation of the search stimulus. Only neurons that produced a significant sound-evoked increase in firing rate were included in the analysis. The best frequency (BF) of a neuron was defined as the pure tone frequency that generated the highest firing rate in the recorded neuron. Best level was defined as the sound level that produced the highest firing rate at BF while threshold was defined as the lowest sound level that produced a firing rate which was at least 2 SD above the spontaneous firing rate. The ability of a neuron to synchronize was calculated using the vector strength (vs) of the response at each modulation frequency $VS=\left(1∕n\right)*\sqrt{\left({\left(\sum \cos\varphi_{i}\right)}^{2}+{\left(\sum \sin\varphi_{i}\right)}^{2}\right)}$, where *n* = total number of observed spikes, Φ*_i_* = phase of observed spike relative to modulation frequency. Statistical significance was assessed using the Rayleigh statistic, to account for differences in the number of driven spikes between neurons, with a Rayleigh statistic value of greater than 13.8 considered to be statistically significant (*p* < 0.001; Mardia and Jupp, 2000).

Neurons were classified as either rate coded, synchronized, or both, depending on changes in firing rate as well as synchrony at different modulation frequencies. Based on rate, the modulation transfer function (rMTF) of a neuron was classified as low-pass (LP), band-pass (BP), all-pass (AP), band-reject (BR) or complex responses. For each neuron, a normalized firing rate was calculated across all the modulation frequencies tested, with the rate at the best modulation frequency (rBMF) denoted as 1, and rates at all other modulation frequencies scaled accordingly. This allowed the comparison of overall shapes of the rMTFs of various neurons with different absolute firing rates. A neuron was classified as BP if the normalized rate dropped below 75% on both sides of the maximum, as LP is the normalized rate dropped below 75% on the high side alone, AP if the normalized rate did not drop below 75% on either side of the maximum and as BR if the normalized rate fell below 66% for a range of modulation frequencies but recovered back up to above 75% on either side. All other neurons were classified as complex responses. The neurons recorded were also divided into synchronous (neurons that represented change in modulation frequency with changes in significant vs) and rate coded (neurons that represented change in modulation frequency with changes in firing rate), with the synchronous neurons classified into low-pass or band-pass based on their temporal modulation transfer function (tMTF) as measured by vector strength (vs) for the synchronous units. In addition, for the synchronous units, a temporal best modulation frequency (tBMF) was calculated as the modulation frequency that produced the greatest vs, and an *F*_max_ was calculated as the highest modulation frequency that synchronized with a Rayleigh statistic greater than 13.8.

### Single compartment IC model

#### Intrinsic properties

Two separate neuron models were developed based on intrinsic properties described in previous intracellular studies (Sivaramakrishnan and Oliver, 2001; Koch and Grothe, 2003; Wu et al., 2004; Tan et al., 2007), and these basic models were modified to produce other observed firing patterns for current injection. The sustained model consisted of a fast transient Na^+^ current (*I*_Na_), a delayed rectifier potassium current (*I*_kDr_), a high-threshold potassium current (*I*_kHT_), and a TEA-sensitive potassium current (*I*_kTEA_). The adapting model consisted of a fast transient Na^+^ current (*I*_Na_), a delayed rectifier potassium current (*I*_kDr_), a TEA-sensitive potassium current (*I*_kTEA_), a low-threshold Ca2+ current (*I*_T_), a high-threshold Ca2+ current (*I*_L_), an apamin-sensitive calcium-activated potassium current (*I*_Sk_), an apamin-insensitive, large conductance Ca^2+^ dependent potassium channel (*I*_Bk_), and a hyperpolarization-activated cation current (*I*_h_). Both models also included synaptic currents (*I*_syn_), an injected current (*I*_inj_), and a leak current (*I*_leak_). Model equations and constants are given in the Appendix.

The leak conductances for each model were adjusted so that their resting membrane potentials were approximately −70 mV. Subsequent small adjustments to membrane potential were done through constant injected current applied throughout the simulation run, at least 200 ms prior to synaptic stimulation, ensuring the membrane potential reaches steady state as noted and placing the membrane potential in the range found *in vivo* (Tan et al., 2007; Geis and Borst, 2009).

#### Synaptic inputs

The inputs to the IC model represented realistic *in vivo* responses from cochlear nucleus (Joris and Yin, 1998; Schatteman et al., 2008), superior olivary complex (Grothe et al., 1997; Joris and Yin, 1998), and lateral lemniscus (Yang and Pollak, 1997; Zhang and Kelly, 2006) to sAM stimuli.

Each individual input to the model IC neuron was modeled as a series of spike times. For a given input, each individual afferent spike train was created based on spike probabilities calculated from rate and temporal MTFs from responses to sAM stimuli in the ascending input sources listed above. The statistical model used to generate these inputs is given in the Appendix. Each trial for a given IC input train had a unique set of generated spike times. To use numbers of trials similar to that collected during typical *in vivo* recordings, 10 trials of a given stimulus were typically used to generate model responses using a given set of parameters. Modulation frequencies ranged from 8 to 1024 Hz in octave steps, similar to the *in vivo* recordings.

The generated input spike times were used to generate the synaptic currents in a method similar to a previous modeling study (Rabang and Bartlett, 2011). Each input spike time would trigger a synaptic event that produces a measured increase in synaptic conductance. The amplitude and time constants used to produce the synaptic events were adjusted to fit amplitude, rise and decay characteristics using data reported from *in vitro* studies of IC synaptic currents and given in the Appendix (Wu et al., 2004). Excitatory inputs consisted of an AMPA and NMDA component and inhibitory inputs consisted of a GABA_A_ current. Synaptic depression of the AMPA and GABA_A_ current was modeled via an interval-dependent amplitude scale factor of the resulting excitatory and inhibitory conductances, which were based on previous intracellular IC studies in rats (Wu et al., 2004; Sivaramakrishnan and Oliver, 2006).

The synaptic input characteristics, input spike times, and synaptic conductance values were written, generated and run through MATLAB (Mathworks, Inc.). Using these parameters, the adapting and sustained IC neuron model simulations were performed in the NEURON simulation environment (Hines and Carnevale, 2001). Analysis was done in MATLAB. All computation and analysis were performed on DELL workstations using the MS Windows 7 operating system. The simulation trials used an integration time step value d*t* = 0.02 ms. This value was empirically verified to be sufficient to simulate accurate ion channel and synaptic currents.

#### User-defined inputs

In addition to using physiologically derived rate and temporal characteristics to generate realistic synaptic inputs, we generated synaptic inputs that were shaped by user-defined input rate and temporal MTFs. We used these user-defined inputs to highlight transformation of input to output responses via the IC models. Excitatory inputs were based on normalized rate curves that were BP, AP, or LP. The tMTF of these inputs were similar to the LSO inputs (Figure 3). User-defined inhibitory inputs were either AP rate with DNLL LP tMTF characteristics, or BP rates generated with user-defined rBMF. Example user-defined shape input MTFs and model responses are shown and used in Figure 3.

#### Model data analysis

Unless otherwise noted, spike counts and firing rates were computed from the entire stimulus duration. Synchrony of the model response was analyzed similarly to the single unit recordings using vs and Rayleigh statistic. The time window for Rayleigh computation was 50 ms following stimulus onset through the duration of the 750 ms stimulus. Output responses were designated as onset or sustained, and either LP, AP, high-pass, BP, or BR by similar criteria used for experimental recordings.

### Immunohistochemistry

The ICs of four young (9–12 weeks) and four aged (92–95 weeks) Fischer-344 rats were processed for immunohistochemistry. Animals were euthanized with Beuthanasia (200 mg/kg). Once areflexive, they were perfused transcardially with 150–200 mL phosphate buffered saline with 0.1% heparin followed by 400–500 mL 4% paraformaldehyde. The brains were removed and stored overnight in 4% paraformaldehyde, after which they were transferred to 30% sucrose for cryoprotection and frozen. Thirty or 35 μm free-floating sections were obtained using a Shandon FE cryotome (Thermo-Electron). Free-floating sections were processed for immuno-histochemical labeling of GAD 65/67 and VGluT2. Rabbit anti –GAD 65/67 (1:1000, Millipore) was used as the primary antibody for GAD 65/67. Guinea pig anti-VGluT2 (1:500, Millipore) was used as the primary antibody for VGluT2. The specificities of these antibodies have been verified in previous studies (Belenky et al., 2008; Cooper and Gillespie, 2011). Biotinylated secondary antibodies (1:200, Vector Laboratories) were used for both. Tissue was then processed using the Vectastain ABC kit (Vector Laboratories), and antibody localization was visualized by DAB (diaminobenzidine). Sections were mounted onto Superfrost Plus slides, dehydrated, and cover slipped using Permount (Fisher). For two sets of the animals (one young and one aged animal being a set), the tissue was processed on the same day with the same reagents. All tissue was stained using aliquots from the same original vial of antibody. For every round of staining, two negative controls were used to verify the specificity of the signal from the oxidized DAB precipitate: (1) no primary antibody and (2) no secondary antibody.

#### Image analysis

ImageJ software (NIH) was used to analyze four random, 1600 μm^2^ regions of interest (ROI’s) from within the IC central nucleus of each selected section at 40× using a Zeiss AxioObserver.Z1. Pixel intensity values were normalized against unstained cells to obtain the relative optical density (ROD). Unstained cells were characterized as such because their average pixel intensities were within one SD of the average pixel intensity values of the deep cortical white matter in the same section. Eight to ten sections per animal were analyzed, so the ROD for each animal was based on the average ROD of 32–40 ROIs. For illustrative purposes, ROD was shown as 1-ROD in figures so that high values correspond to dark pixel intensities.

## Results

### Responses of neurons in the IC of young animals to AM stimuli

Responses from 92 auditory neurons were recorded from the IC of four young animals. Of these, 37 (40%) neurons had non-synchronized, purely onset responses to AM stimuli that were not modulated by modulation frequency, and were discarded for the purposes of this study. These neurons were often encountered early in a dorso-ventral oriented track and were likely to be located in dorsal or external cortex of IC. The remaining neurons all responded to varying modulation frequencies either by change in rate or synchrony. Of these, the percentages of neurons exhibiting BP and LP rMTFs were 24% (*n* = 13) and 36% (*n* = 20), respectively. Forty percentage of the neurons not classified as either BP or LP rMTFs were distributed equally into AP (*n* = 11) and BR (*n* = 11) rMTFs with a few neurons (*n* = 4) showing complex rate tuning (see Materials and Methods for definitions). A small number (*n* = 3) of the neurons exhibited sustained discharges but were not synchronized to any modulation frequency and represented changes in modulation frequency purely by changes in rate. These neurons were not used in for the analysis of synchrony. Of the synchronized neurons, almost equal numbers exhibited LP (56%, *n* = 31) and BP (44%, *n* = 24) behavior in their tMTFs.

### Single compartment IC model – membrane and synaptic properties

**Figure 1:**
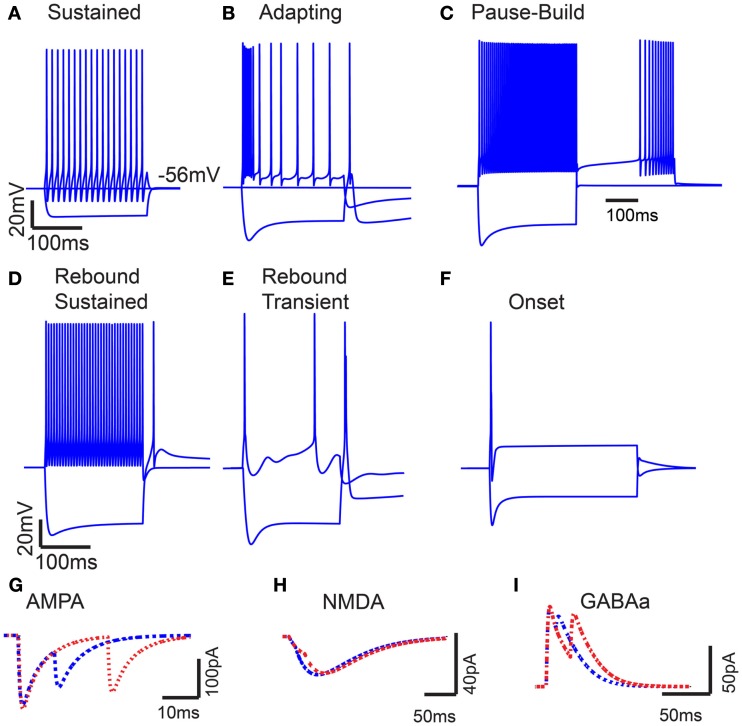
**Model intrinsic and synaptic properties**. **(A)** IC Sustained model response to 200 pA depolarizing and hyperpolarizing current pulses. Voltage traces measured in mV (*Y*-axis) plotted versus time in ms (*X*-axis). **(B)** Adapting model response to 300 pA depolarizing and hyperpolarizing current pulses. **(C)** Pause-build responses to a 500 pA depolarizing pulse and a negative 500 pA hyperpolarizing current pulse, followed by a 500 pA depolarizing current pulse. **(D)** Rebound-Sustained response to 500 pA depolarizing and hyperpolarizing current pulses. **(E)** Rebound-Transient response to 400 pA depolarizing and hyperpolarizing current pulses. **(F)** Onset response to 800 pA depolarizing and hyperpolarizing current pulses. *Y*-axis represents voltage traces measured in mV and *X*-axis represents time in ms for **(A–F)**. **(G–I)** Current traces of paired post-synaptic AMPA, NMDA, and GABA_A_ currents, respectively. *Y*-axis represents synaptic current in nA, and *X*-axis represents time ms. Paired inputs at 10 *(blue)* and 25 *(red)* ms ISI to show time course and magnitude of synaptic depression.

Two basic single compartment models were created to simulate sustained and adapting firing patterns in IC (Figures 1A,B), which are the two most commonly observed firing patterns in response to current injection *in vivo* (Tan et al., 2007) and *in vitro* (Sivaramakrishnan and Oliver, 2001). The Sustained model produced regular firing to depolarizing current injections. The Adapting model produced a higher frequency onset burst followed by sustained spiking with rate adaptation to depolarizing currents. A low-threshold Ca^2+^ dependent current (*I*_T_) produced rebound depolarization following hyperpolarization, and *I*_h_ was included for this model because it is commonly found in adapting neurons (Tan et al., 2007). Figures 1A,B shows the model responses to depolarizing and hyperpolarizing current pulses for Sustained and Adapting models, respectively. Each model had a resting membrane potential of −70 mV; a bias current was applied during each simulation to adjust membrane potential to between −64 and −56 mV, which accounts for mean depolarization provided by ongoing synaptic activity. This is within range of typical values recorded from IC neurons *in vivo* (Tan et al., 2007; Geis and Borst, 2009). The Sustained and Adapting model had calculated membrane resistances of 146 and 142 MΩ and a time constant of 4.4 and 5 ms, respectively, in line with previous measurements (Tan et al., 2007).

The basic Sustained and Adapting models were then elaborated to create Pause-Build (Figure 1C), Rebound-Sustained (Figure 1D), or Rebound-Transient (Figure 1E) models corresponding to response classes reported in previous studies (Sivaramakrishnan and Oliver, 2001; Tan et al., 2007). For the Pause-Build model, a rapidly inactivating potassium conductance was added, along with a small *I*_h_ current, while the high-threshold potassium conductance was removed and the low-threshold potassium and leak conductances were lowered (Appendix). While this did not affect responses to depolarizing current pulses, the presence of the A-type potassium conductance produced a delayed onset to firing when a depolarizing current followed hyperpolarization that deinactivated the potassium conductance (Figure 1C). The Rebound-Sustained model is the Sustained model with the presence of a T-type calcium conductance (Figure 1D), which affected responses mainly when hyperpolarized by inhibition or neuromodulators. To obtain the Rebound-Transient response, the Adapting model was adjusted by increasing the L-type calcium conductance and reversing the relative strengths of the SK and BK calcium-activated potassium conductances. In addition to producing transient burst responses (Figure 1E), these adjustments also produced depolarizing afterpotentials (not shown), as reported in Sivaramakrishnan and Oliver (2001). Onset responses to current injection have been reported as a small proportion of responses (Sivaramakrishnan and Oliver, 2001; Bal et al., 2002) or absent (Tan et al., 2007) in previous studies. The Onset model was configured differently than the Sustained or Adapting models. The Onset model contained a transient sodium, potassium delayed rectifier, low-threshold potassium (Rothman and Manis, 2003), and a hyperpolarization-activated cation current (Koch and Grothe, 2003). The conductances for each were adjusted to fit firing patterns to depolarizing current pulses (Sivaramakrishnan and Oliver, 2001). Onset model responses produced a single spike for depolarizing current pulses (Figure 1F). Because the Adapting and Sustained responses comprised the majority of IC responses, these have been used for the remainder of the results, leaving exploration of the interaction between subtype membrane and synaptic properties for future study. Adapting and Sustained model responses to identical stimuli typically produced similarly shaped rMTFs, although Sustained model responses exhibited higher rates overall at the same membrane potential. This is likely due to the larger membrane resistance in the Sustained model and the addition of potassium currents in the Adapting model. As a result, Sustained model responses often had lower vs values due to a larger number of action potentials observed within a given period and greater sensitivity to small membrane potential fluctuations. As the name implies, there was a gradual decline in firing rate for sustained synaptic stimulation in the Adapting model but not in the Sustained model.

Excitatory synaptic inputs consisted of AMPA and NMDA receptor components. Inhibitory inputs consisted of a GABA_A_ receptor component. Both AMPA and GABA_A_ components exhibited short-term synaptic depression, which was modeled from *in vitro* voltage clamp studies in rat IC (Wu et al., 2004). Example post-synaptic currents with pairs of inputs at 10 and 25 ms ISI are shown for each synaptic current on Figures 1G–I.

### Rate and temporal characteristics of model inputs

**Figure 2:**
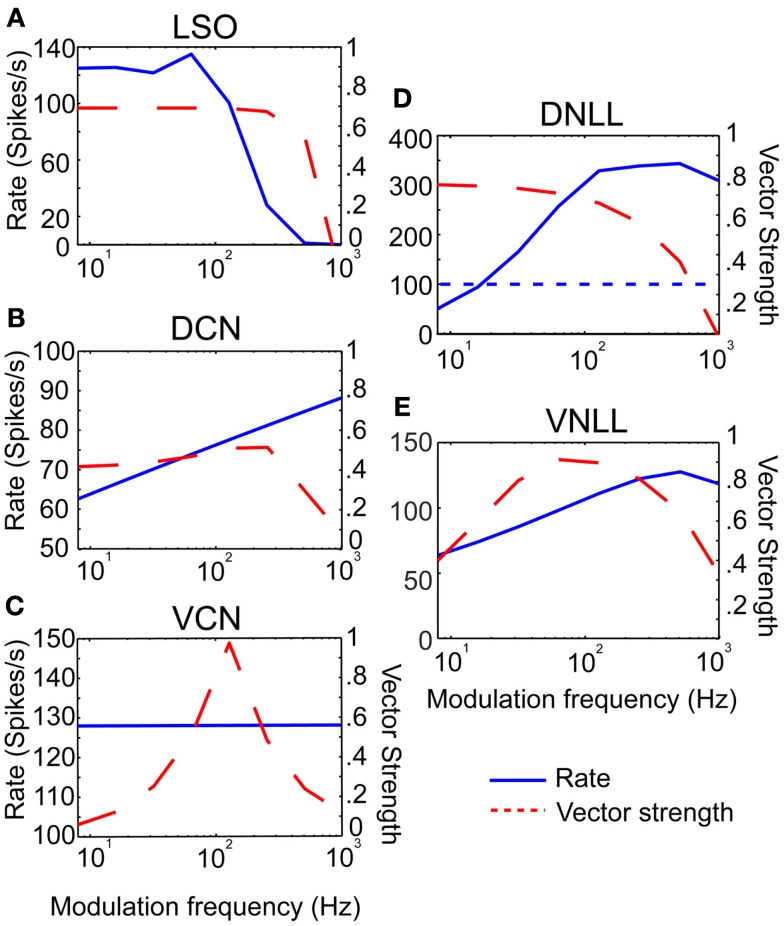
**Excitatory and inhibitory model input characteristics**. **(A–C)** Rate in spikes/s *(blue)* and vector strength *(red)* curves characterizing inputs from LSO, DCN, and VCN, respectively. Rate and vector strength are marked on opposing *Y*-axes. Modulation frequency is marked along the *X*-axis in Hz. **(D–E)** Rate and vector strength curves characterizing inhibitory inputs from DNLL and VNLL. DNLL inputs either had all-pass or high-pass rate characteristics, as shown. Axes similar to **(A)**.

Three example excitatory input sources were chosen for synaptic input to the IC model; LSO, DCN, and VCN (Cant and Benson, 2006), and were also used as a means to represent different rate tuning shapes of physiological inputs to IC. The input rMTF and tMTFs for each example input are given in Figures 2A–C. DCN input rates (Schatteman et al., 2008) increased monotonically but over a relatively small range of rates with increasing modulation frequency. LSO input rates (Joris and Yin, 1998) were LP with peak rates and tMTF cut-off at 64 Hz. VCN input rates (Joris and Yin, 1998) were AP tuned. The tMTF curves for the DCN and LSO inputs were all LP. VCN input synchrony was BP tuned to 128 Hz, simulating the response to suprathreshold intensities (Møller, 1972; Frisina et al., 1990) while the rate tuning was flat for all modulation frequencies. These examples were culled from previous literature, but are not intended to represent the full response distributions of any given afferent to IC. Values for a given class of inputs could be adjusted easily to fit individual examples, if desired.

Two inhibitory input sources were used: DNLL and VNLL (Cant and Benson, 2006). The input rMTF and tMTFs are shown on Figures 2D,E. DNLL input rates were either high-pass (Figure 2D) or AP tuned, with LP synchrony. VNLL input rates were either BP or high pass with BP synchrony tuned between 64 and 128 Hz (Figure 2E). Each MTF was modeled from physiological data (Yang and Pollak, 1997; Zhang and Kelly, 2006).

### Comparison of model input and output rate and synchrony

**Figure 3:**
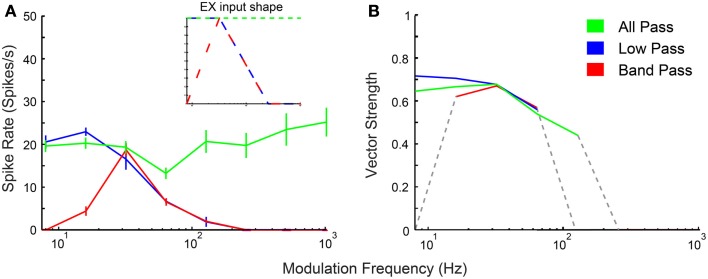
**IC model responses to general AM rate tuning of inputs**. User-defined all-pass *(green)*, low-pass *(blue)*, and band-pass *(red)* excitatory inputs. Three excitatory inputs with LSO tuned synchrony coupled with two DNLL all-pass inhibitory inputs using the Adapting model. **(A)** Rate response shapes reflect input rate characteristics. Firing rate in spikes/second is marked along the *Y*-axis and modulation frequency in Hz is marked along the *X*-axis. *Inset:* Input rate shape characteristics. **(B)** Vector strength responses to user-defined inputs. Vector strength responses that only show significant synchrony (Rayleigh Stat >13.8, *p* < 0.001) are displayed. Vector strength is marked along the *Y*-axis and modulation frequency in Hz is marked along the *X*-axis.

In addition to physiologically derived input characteristics from previous studies, user-defined tuning shape input rates, and temporal MTFs were also produced to highlight the transformation of input to output responses via the IC models. By creating generalized LP, BP, and AP rate tuning shapes, it was easier to isolate transformations in responses generated by IC synaptic and membrane characteristics. User-defined EX inputs were based on normalized rate curves that were BP, AP, or LP. The EX inputs used a LP tMTF similar to the LSO inputs. User-defined IN inputs were either AP rate with DNLL LP tMTF characteristics, or BP rate with user-defined rBMF. Example model responses to EX inputs with corresponding input MTFs are shown in Figure 3 for an Adapting neuron model. BP EX inputs typically produced similar model output rate responses, while LP and AP inputs produced nearly all LP rate responses. Similar results were observed for the Sustained model.

### Simulation of example *in vivo* response types using single compartment model

Detailed response characteristics of example neurons are presented below. These were selected to represent neurons from four broad categories of rate coding – BP, LP, BR, and AP. The responses of these neurons with respect to rate and synchrony are described below and the parameters of the model that were modified to produce these specific neuronal types are also described. By changing a few physiologically relevant factors in the model, more than 90% of the neuronal response types encountered in the IC to AM stimuli were reproduced.

#### Low-pass rate responses

**Figure 4:**
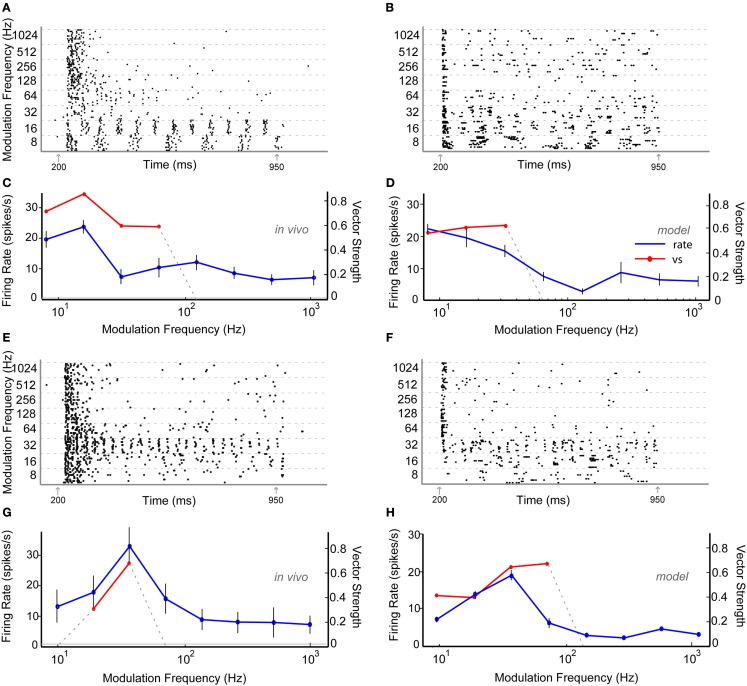
**Example *in vivo* and model neurons exhibiting either low-pass rMTF (A–D) or band-pass rMTF (E–H)**. **(A)** Dot-raster plot of responses of unit Y27 from the IC of a young animal to nAM stimuli presented at 65 dB SPL. For this example and other raster examples, modulation frequency is marked along the *Y*-axis in Hz and time along the *X*-axis in ms. Each black dot represents a single action potential. Gray arrows indicate beginning and end of sound stimulus. Each modulation frequency shows an aggregation of 10 trials. **(B)** Dot-raster plot of an Adapting model neuron using 2 VCN and 4 high-pass rate tuned DNLL inputs. **(C)** Mean firing rate and vector strength of neuron shown in **(A)** for various modulation frequencies, exhibiting low-pass rMTF and tMTF. Rate in spikes/s *(blue)* and significant vector strength *(red)* are marked on opposing *Y*-axes. Modulation frequency is marked along the *X*-axis in Hz. Horizontal gray line indicates mean spontaneous firing rate. Error bars indicate 1 standard deviation from mean firing rate. **(D)** Adapting IC model neuron using inputs described in **(B)** showing mean firing rate (blue) of 10 trials, and significant vector strength (red). Axes similar to **(C)**. **(E)** Dot-raster plot of responses of unit Y30 from the IC of a young animal to nAM stimuli presented at 75 dB SPL. **(F)** Dot-raster plot of a Sustained model neuron using three user-defined low-pass excitatory inputs with six low-pass inhibitory inputs, each with corner frequencies at 32 Hz. **(G)** Mean firing rate and vector strength of neuron shown in **(E)** for various modulation frequencies, exhibiting band-pass rMTF and tMTF. Axes similar to **(C)**. **(H)** Mean firing rate and vector strength of model neuron described in **(F)** for various modulation frequencies, showing mean firing rate (blue) of 10 trials, and significant vector strength (red). Axes similar to **(G)**.

Thirty six percentage of the neurons recorded *in vivo* exhibited LP rMTFs as described in the Section “Materials and Methods.” These LP rate responses typically produced rBMFs of 8–16 Hz, with the responses dropping below 75% (*F*_max_) at frequencies from 32 to 128 Hz. These neurons also exhibited synchrony in a LP (60%) or BP (40%) manner, with the rBMFs and tBMFs typically differing for both response sub-types. Figures 4A,C shows a representative LP rate coded unit, Y27, which exhibited LP rMTF and tMTF for nAM stimuli presented at 65 dB SPL. In this example, the rBMF and the tBMF were both 16 Hz, with a rate of 23.7 spikes/s and vs of 0.85.

Low-pass model responses were most commonly generated with LP or AP excitatory inputs (i.e., LSO, VCN, user-defined EX inputs) coupled with inhibitory high-pass or AP inputs (VNLL, DNLL). Nearly all LP model rate responses produced a LP tMTF. An Adapting model response to AP VCN excitatory and high-pass DNLL inhibitory inputs shows an example of LP rate and synchrony (Figures 4B,D).

#### Band-pass rate tuning

Twenty-four percentage of the neurons encountered in young animals exhibited BP rMTFs. Of these, a majority of the neurons also exhibited BP coding for synchrony. The rBMF and the tBMF were the same for all but one neuron in this category. Figures 4E,G shows a representative neuron, Y30, exhibiting BP rate and synchrony coding to nAM stimuli presented at 75 dB SPL. In this example, the neuron exhibited strong onset responses and a few scattered action potentials that changed only minimally at all modulation frequencies except at 16–32 Hz, with a BMF of 32 Hz. At 32 Hz, the neuron strongly phase-locked to the modulation frequency, producing a concurrent increase in rate. The vs at BMF was 0.67, and the peak firing rate was 32.9 spikes/s.

**Figure A2:**
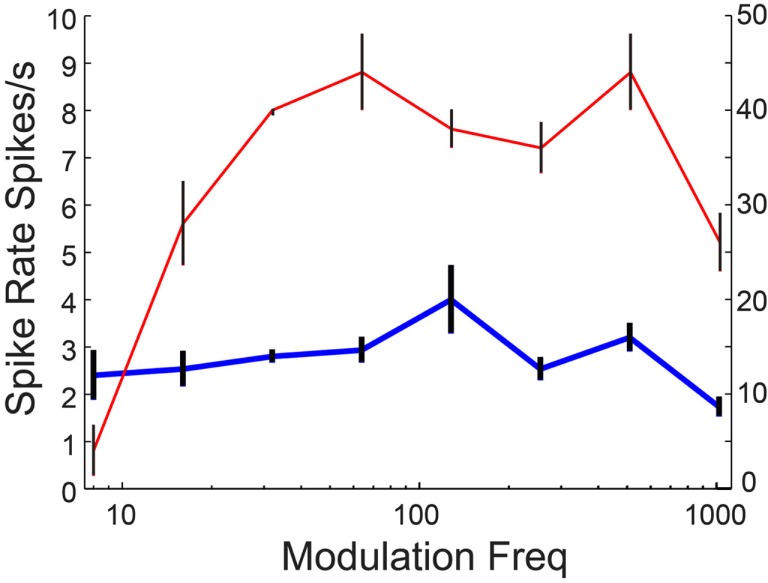
**Band-pass responses using excitatory inputs**. Blue line shows rate responses taken from 750 ms stimulation duration (Y-axis scale, left). Red line shows rate responses taken from first 50 ms of stimulus onset (Y-axis scale, right).

Model BP responses could be generated using excitatory inputs that also were BP tuned (i.e., BP EX inputs, Figure 3), LP excitatory inputs coupled with BP inhibitory inputs tuned to lower (<64 Hz) frequencies (Figure 11) or LP excitatory inputs and LP inhibitory inputs with equal or higher corner frequency for the excitatory inputs (Figures 4F,H, Dicke et al., 2007). Synaptic inhibition is not necessarily required for BP rate tuning. However, preliminary testing suggests that other mechanisms that may produce BP mechanisms from only excitatory inputs (e.g., coincidence detection) are difficult to make compatible with the combination of input firing rates, membrane properties, and time constants, and synaptic properties and time constants. We did produce BP responses using only excitatory inputs. However, these were only produced using a limited set of parameters (Figure A2 in Appendix). Figures 4F,H shows model BP rate and synchrony responses using LP excitatory inputs with LP inhibition with corner frequencies at 32 Hz.

A small number of neurons (*n* = 4) that exhibited BP rate coding also exhibited a LP coding for synchrony. This was reproduced in the model using low-pass LSO excitatory inputs and BP VNLL inputs (not shown).

#### All-pass, band-reject, and other rate responses

Forty percentage of the neurons encountered did not exhibit BP or LP rate tuning. Of these, half the neurons exhibited AP rate tuning and did not change in rate significantly with changes in modulation frequency, but selectively encoded these changes in modulation frequencies by changes in synchrony and in some cases changes in overall firing pattern. Another 36% of these units exhibited BR responses, where their normalized rates dropped below 67% for a small range of modulation frequencies but were flanked by higher rates for lower and higher modulation frequencies. A small number (*n* = 4) also exhibited other rate responses, consisting of high-pass, double-peaked, or complex tuning.

Of these units that exhibited AP, BR or other rate response types, 68% exhibited LP tMTF characteristics and 32% exhibited BP tMTF characteristics. The tBMFs of these units typically ranged from 8 to 64 Hz, and the cut-off frequencies ranged from 16 to 256 Hz.

**Figure 5:**
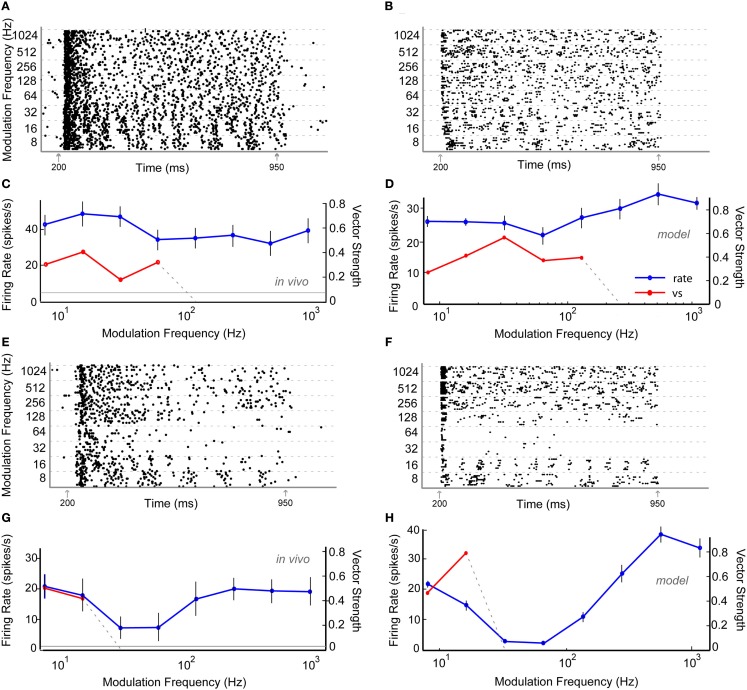
**Example *in vivo* and model neurons exhibiting all-pass rMTF and low-pass tMTF (A–D) or band-reject rMTF and low-pass tMTF (E–H)**. **(A)** Dot-raster plot of responses of unit Y34 from the IC of a young animal to nAM stimuli presented at 65 dB SPL. **(B)** Dot-raster plot of an Adapting model neuron using three DCN and two all-pass rate tuned DNLL inputs. **(C)** Mean firing rate and vector strength of neuron shown in **(A)** for various modulation frequencies, exhibiting all-pass rMTF and low-pass tMTF. **(D)** Mean firing rate and vector strength of the model neuron described in **(B)**. **(E)** Dot-raster plot of responses of unit Y22 from the IC of a young animal to nAM stimuli presented at 65 dB SPL. **(F)** Dot-raster plot of responses from an Adapting model neuron with 2 VCN and 6 VNLL band-pass rate tuned inputs (rBMF = 36 Hz). **(G)** Mean firing rate and vector strength of neuron shown in **(E)** for various modulation frequencies, exhibiting band-reject rMTF and low-pass tMTF. **(H)** Mean firing rate *(blue)* of 10 trials, and significant vector strength *(red)* of model neuron shown in **(F)**. All axes similar to Figure 4.

Figures 5A,C shows a representative neuron, Y34, which exhibited AP rate coding and a LP synchrony coding to sAM stimuli centered at 1.7 kHz presented at 65 dB SPL. The tBMF was 16 Hz with vs of 0.41 and the mean firing rate was 39.3 ± 5.9 spikes/s. AP model responses were generated using excitatory AP (VCN) or high-pass rate responses (DCN) with inhibitory high-pass or AP rate inputs (DNLL). These responses also produced a LP tMTF. An Adapting model response using DCN and DNLL inputs is shown on Figures 5B,D.

Figures 5E,G shows a representative neuron, Y22, exhibiting BR rate coding and LP synchrony coding to nAM stimuli presented at 65 dB SPL. In this example, the normalized rate dropped below 67% at 32 and 64 Hz AM, while staying above 75% on either side. The tBMF for this unit was 8 Hz, with vs of 0.50.

**Figure A3:**
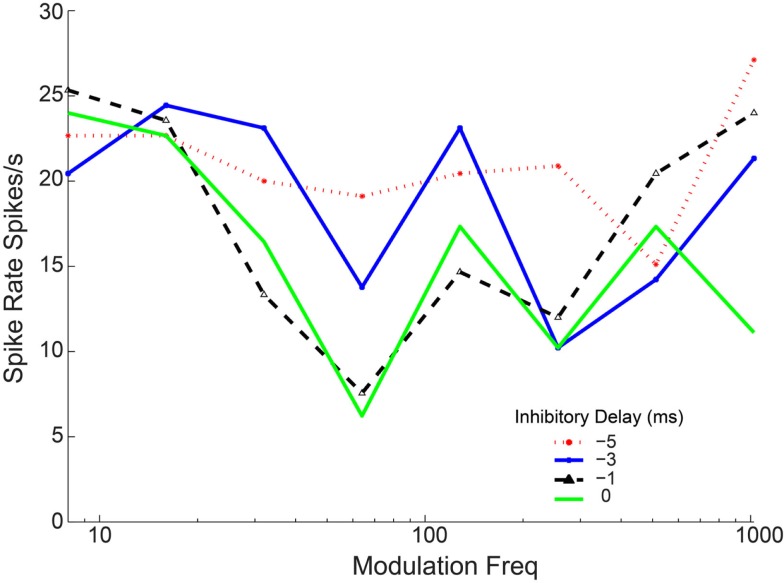
**Inhibition timing can affect rMTF response shape**. Three VCN excitatory and three DNLL all-pass tuned inhibitory inputs to an Adapting model. Inhibitory inputs adjusted such that they are conincident (green) or precede excitation by 1, 3, or 5 ms (black, blue, red lines, respectively).

Band-reject rate coded model responses were produced using BP inhibitory inputs with VNLL synchrony coding. The BR region of the output response was typically produced at or within an octave range of the BP inhibitory input rBMF. BR rate coded responses were also created using inhibitory inputs that preceded excitatory inputs for a limited set of parameters (Figure A3 in Appendix). The example in Figures 5F,H shows an Adapting model response to AP VCN excitatory inputs with BP VNLL synchrony coded inhibitory inputs with tBMF at 36 Hz.

### Determinants of output tMTF

*In vitro* studies suggest a moderate number of inputs (1–10) converge on IC neurons (Smith, 1992; Wu et al., 2004). The balance of excitation and inhibition has varied substantially between IC models, ranging from no inhibition (Hewitt and Meddis, 1994) to dominant inhibition (Nelson and Carney, 2004). Using two to five excitatory and inhibitory inputs, with combinations where the number of excitatory and inhibitory inputs were nearly balanced or were dominated by inhibition, produced the best results. In terms of membrane potential fluctuations, we found that smaller numbers of excitatory inputs (2–3) produced more phasic and peaked membrane fluctuations for each stimulus modulation period, whereas larger numbers of excitatory inputs (7–9) generated smoother membrane potential fluctuations that followed the amplitude modulation contour (Figures 7G–I, number of inhibitory inputs fixed at five). These membrane potential fluctuations are similar to those described by Geis and Borst (2009). Other studies have also assumed inhibition would lag excitation (∼1 ms; Cai et al., 1998a,b; Borisyuk et al., 2002). However, our model does not show strong qualitative differences in responses to shifts of inhibition timing relative to excitation (±5 ms, data not shown), likely owing to multiple inputs with multiple spikes per cycle at lower modulation frequencies which renders delay less meaningful after the initial response.

**Figure 6:**
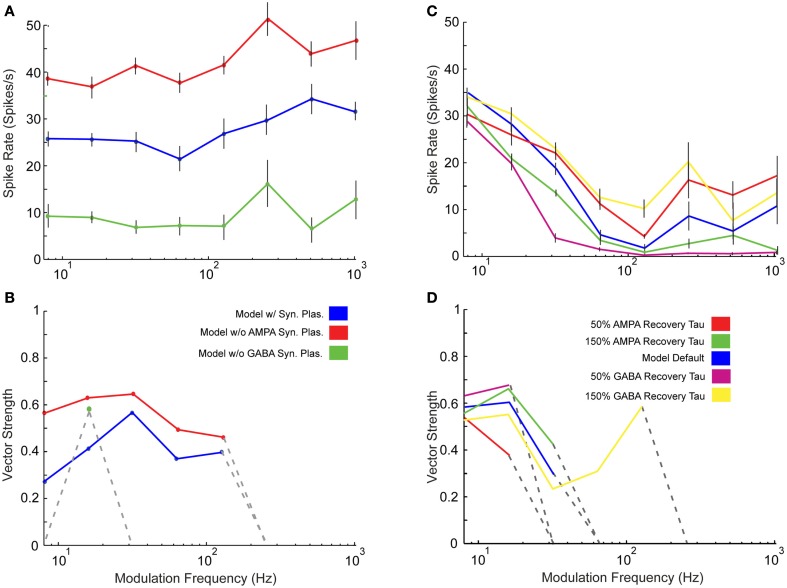
**Effects of synaptic plasticity on model responses**. Changes in rate **(A)** and statistically significant vector strength **(B)** with modulation frequency in IC Adapting Model responses with 3 DCN and 2 DNLL all-pass inputs in the presence of synaptic depression *(blue)*, the absence of only AMPA synaptic depression *(red)* or the absence of only GABA_A_ synaptic depression *(green)*. Changes in rate **(C)** and statistically significant vector strength **(D)** responses with modulation frequency in IC Adapting model with 2 VCN and 4 DNLL high-pass inputs under normal GABA_A_ and AMPA recovery time constants *(blue)*, 50% reduction in AMPA *(red)* or GABA_A_ *(purple)*, or 150% increase in AMPA *(green)* or GABA_A_ *(yellow)* time constants. Synchrony range is increased with longer recovery time but decreased with shorter recovery time. Modulation frequency in Hz is marked along the *X*-axes for **(A–D)**, mean firing rate in spikes/s is marked along the *Y*-axes in **(A,C)** and vector strength is marked along the *Y*-axes in **(B,D)**.

One feature of this IC model is the use of short-term synaptic depression measured in previous *in vitro* studies (Wu et al., 2004). We compared a limited set of model responses with and without the presence of synaptic depression in the AMPA and GABA_A_ receptor mediated currents. A noticeable change in rate was observed as responses without AMPA plasticity increased at nearly all modulation frequencies (Figure 6A). There were few qualitative changes in vs or changes in synchrony cut-off (Figure 6B), so AMPA plasticity under these conditions made these responses more energetically efficient, meaning that the same features could be represented with far fewer spikes, which are energetically costly (Attwell and Laughlin, 2001). Removing GABA_A_ plasticity, however, reduced rate responses at all modulation frequencies (Figure 6A). In addition, synchrony cut-off was reduced due to low spike output (Figure 6B).

The model recovery time constant from synaptic depression was also varied by changing the value of TauR2 for AMPA and TauR for GABA_A_ synaptic currents (Table A4 in Appendix). Either increasing or decreasing the depression recovery time by 50% for either the AMPA or GABA_A_ plasticity produced similar rate responses at 8 Hz (Figure 6C). For higher modulation frequencies, differences were more evident, such as the five-fold difference in firing rates at 32 Hz between 50% GABA recovery tau versus 150% recovery tau. The range of synchrony was decreased when AMPA or GABA_A_ recovery time was halved, while increasing the recovery time for GABA_A_ increased synchronized response range (Figure 6D).

**Figure 7:**
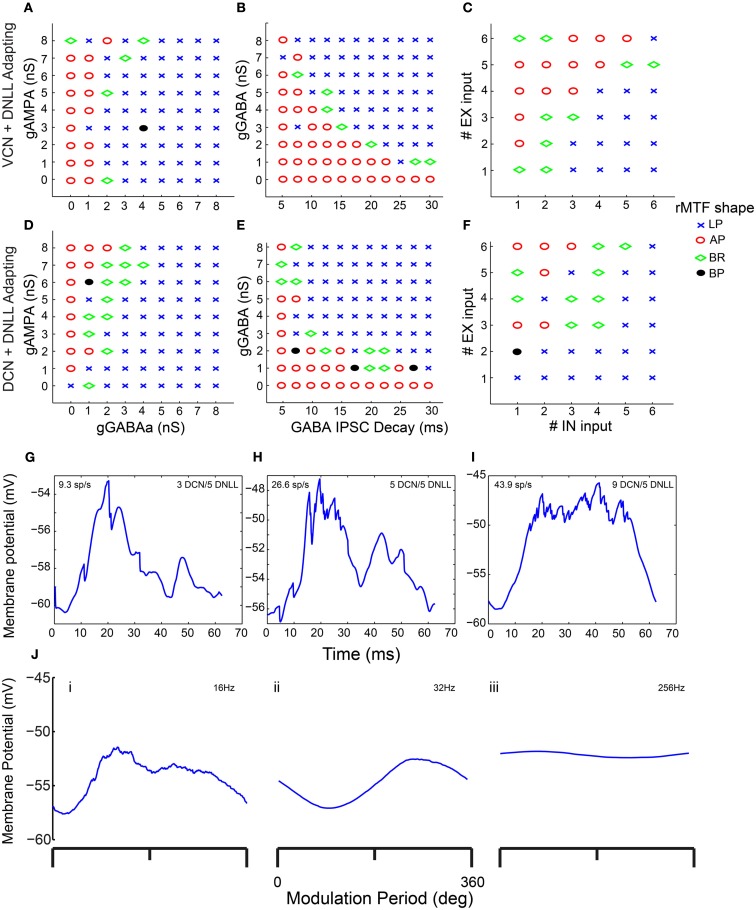
**Determinants of IC Model output and rMTF shape**. Color plots showing various rate MTF response shapes using 4 VCN excitatory and 4 DNLL high-pass inhibitory **(A–C)** inputs with the IC Adapting model. LP – low-pass *(blue crosses)*, AP – all-pass *(red circles)*, BR – band-reject *(green diamonds)*, BP – band-pass *(black circles)*. **(A)** Rate shape plots as a result of adjusting GABA_A_ synaptic conductance (nS, *X*-axis) and AMPA synaptic conductance (nS, *Y*-axis). **(B)** Rate shape plots corresponding to changes in GABA_A_ ISPC decay (ms, *X*-axis) and GABA_A_ synaptic conductance (nS, *Y*-axis) **(C)** Rate shape plots corresponding to changes in number of inhibitory (*X*-axis) and excitatory (*Y*-axis). **(D–F)** Color plots similar to **(A–C)**, using 4 DCN and 4 DNLL high-pass inputs in the Adapting model. **(G–I)** Voltage traces taken from a single trial, divided into 62.5 ms intervals (16 Hz) and averaged. Mean voltage trace from a trial using 3 DCN and 5 DNLL high-pass inputs **(G)**, 5 DCN and 5 DNLL high-pass inputs **(H)**, or 9 DCN and 5 DNLL high-pass inputs **(I)**. Membrane potential is marked along the *Y*-axis in mV and time is marked along the *X*-axis in ms. **(J)** Voltage traces taken from a single trial, divided into either 62.5 ms (16 Hz, i), 15.6 ms (64 Hz, ii) or 3.9 ms (256 Hz, iii) intervals and averaged. Mean voltage traces using 3 DCN and 5 DNLL high pass inputs. Membrane potential is marked along the *Y*-axis in mV and modulation period is marked along the *X*-axis in degrees.

In addition to synaptic depression, other potentially important determinants of IC responses were tested in combination with one another to observe their effects on rMTF, including the conductances of AMPA and GABA_A_, the GABA_A_ inhibitory post-synaptic current (IPSC) decay time constant, and the number of excitatory and inhibitory inputs. Each of these parameters can influence the shape of the output rMTF. These values were adjusted in the model, and the output rMTFs were recorded. The summaries of the IC rMTF shapes are shown in Figure 7, using the rMTF shape definitions described in the Section “Materials and Methods.” 4 VCN (Figures 7A–C) or 4 DCN (Figures 7D–F) excitatory inputs and 4 DNLL high-pass inhibitory inputs were used. Model simulations using large GABA_A_ conductances, large IPSC decay values, or large numbers of inhibitory inputs typically produced LP rate responses (blue x), while smaller inhibitory conductances, IPSC decay values or number of inhibitory inputs typically produced AP rate responses (red circles), which are indicated on the color plots (Figures 7A–F). BR responses were produced at intermediate values that separated regions of AP and LP rate responses (green diamonds). BP responses were not commonly observed using the combinations of either DCN/DNLL or VCN/DNLL inputs tested in these examples (black filled circles).

### Anatomic changes in synaptic markers in aged IC

**Figure 8:**
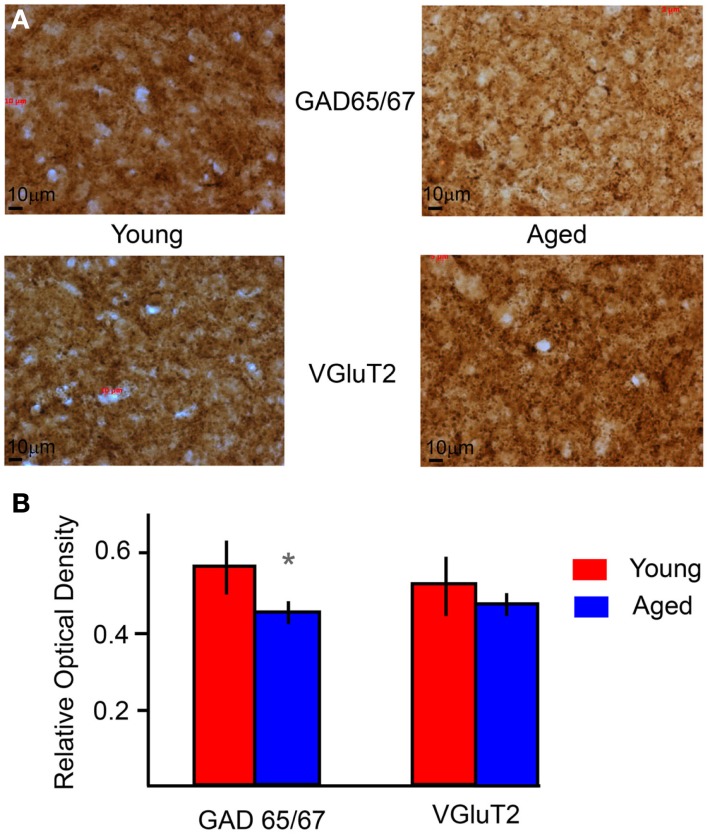
**Immuno-histochemical levels of GAD and VGluT2 in young and aged IC**. **(A)** Example sections of the IC stained for GAD (top) and VGluT2 (bottom) for young (left) and aged (right) animals under 40× magnification. The dimensions of the sections are 208.2 × 156 μm. **(B)** Average relative optical densities of GAD 65/67 and VGluT2 from the IC of young (red) and aged (blue) animals. ROD is marked along the *Y*-axis, and the type of marker along the *X*-axis. Error bars indicate one standard deviation. Asterisks indicate significant difference (*p* < 0.01, unpaired *t*-test).

A main goal in developing the IC model was to apply it to understanding central mechanisms of age-related decline in temporal processing (Schatteman et al., 2008; Parthasarathy and Bartlett, 2011). As a preliminary step to constrain potential mechanisms to test in the IC model, anatomic techniques were used to measure changes in the inhibitory and excitatory synaptic markers. The relative optical densities (RODs) of ROI in the IC central nucleus were compared between young and aged animals for sections immunostained for GAD65/67 a marker of presynaptic GABAergic axons or VGluT2 a marker of a population of glutamatergic axons (e.g., Figure 8A). GAD65/67 ROD showed a significant decrease in aged animals (Figure 8B, *p* < 0.05, unpaired *t*-test), consistent with previous studies showing a loss of inhibitory markers (Raza et al., 1994; Burianova et al., 2009). VGluT2 did not change significantly with age (Figure 8B), consistent with previous studies showing no loss in the number of excitatory terminals in the aged IC (Helfert et al., 1999), though the functionalities of the terminals and post-synaptic receptors are not known. These anatomical data suggested some data-constrained variables suitable for further exploration in the IC model.

### Comparison of *in vivo* responses from the IC of young and aged animals

Responses of 47 auditory neurons were recorded from the IC of three aged animals. Of these, 25 neurons had non-synchronized, purely onset responses to AM stimuli that were not modulated by modulation frequency. These neurons, encountered early in the track and likely to be located in the dorsal or external cortex of the IC, were discarded for the purposes of this study. The remaining neurons all responded to varying modulation frequencies by changes in rate and/or synchrony and were compared to responses obtained from the young animals. Of these neurons, 46% exhibited LP, 36% exhibited BP, and 18% exhibited AP rate tuning. No BR neurons were encountered in our aged sample. The proportion of neurons exhibiting LP characteristics compared to the other response types was slightly higher in the aged than the young. Of the synchronous neurons, the relative proportions of LP and BP tMTFs did not change significantly with age, with 58% exhibiting LP synchrony, and 42% exhibiting BP synchrony.

**Figure 9:**
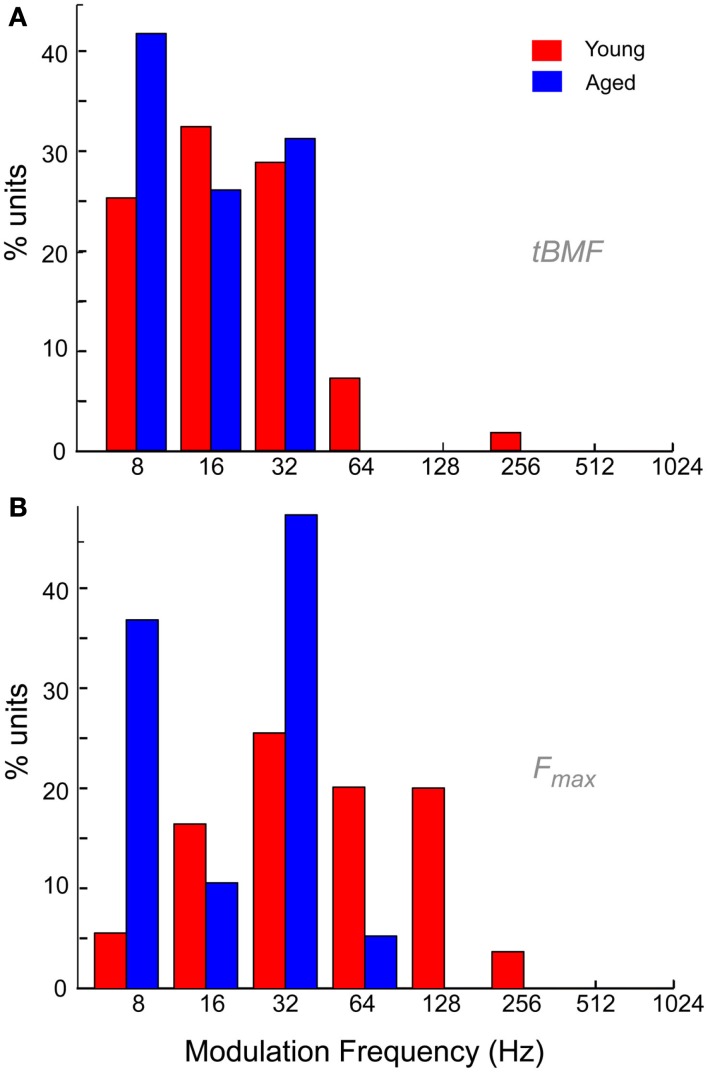
**Temporal characteristics of IC neurons from young and aged animals**. **(A)** Distribution of best temporal modulation frequencies (tBMFs) in young (red) and aged (blue) animals among the recorded neurons in this study. Modulation frequency is marked along the *X*-axis in Hz and *Y*-axis indicates percentage of units with specified tBMF. **(B)** Distribution of temporal cut-off frequencies (*F*_max_) in young (red) and aged (blue) animals among the neurons recorded in this study. Axes same as **(A)**.

The distribution of tBMFs was LP in nature for both young and aged animals. Aged neurons generally had lower vs, and no aged neurons with tBMFs greater than 32 Hz were encountered (Figure 9A). The mean vs at tBMF was also significantly lower for in the aged (0.45 ± 0.12) compared to the young (0.56 ± 0.16, *p* < 0.01, rank-sum test). Figure 9B shows the distribution of *F*_max_ for the young and aged animals. The *F*_max_ or the cut-off frequencies were more band-pass in nature for the young, with the most number of units having a maximum phase locking from 32 to 64 Hz. In the aged, the distribution of *F*_max_ revealed no neurons that could synchronize to MFs greater than 64 Hz AM. The population of neurons that could synchronize to modulation frequencies of 64 Hz and higher, were significantly different between the young and the aged (52.7 vs. 5.3%, *p* < 0.001, χ^2^ test). Thus overall, the aged neurons seemed to lack the ability to synchronize to faster modulation frequencies, and also showed more LP rate tuning and no BR rate tuning.

### Modeling of aged units based on *in vivo* responses

**Figure 10:**
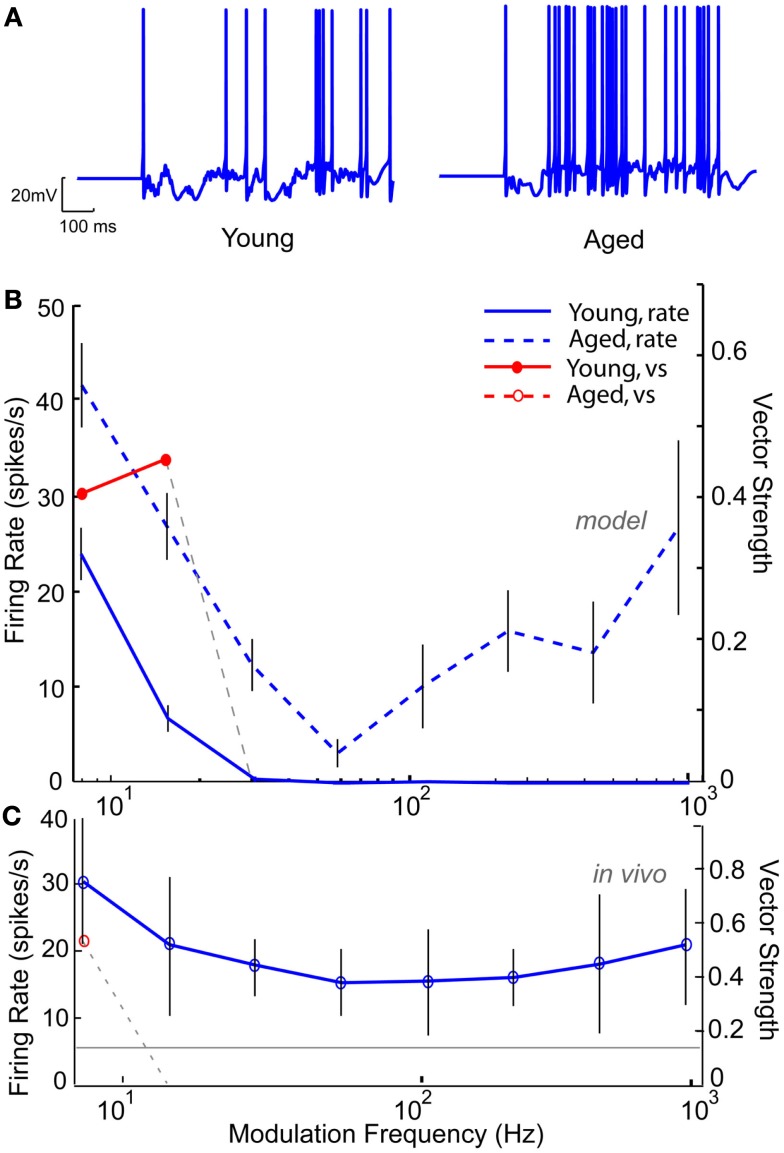
**Modeling age-related loss of synchrony through reduction in GABA_A_**. IC Sustained model with two LSO inputs and three DNLL all-pass and the membrane potential at −60 mV. **(A)** Example voltage traces from individual trials at 16 Hz modulation frequency for a young (left) and aged (right) model neuron. **(B)** Changes in mean firing rate shown for a young model neuron (blue solid line) and an aged model neuron with 50% reduction in GABA_A_ (blue dashed lines). Significant vector strengths shown for a young model neuron (*filled red circles*) and an aged model neuron (*red open circles*, not present in the model). Firing rate, in spikes/s, and vector strength are marked along opposing *Y*-axes and modulation frequency in Hz is marked along the *X*-axis. Error bars indicate one standard deviation from the mean of 10 trials. **(C)** Representative neuron A15 from an aged animal showing a weak low-pass rMTF (*blue solid lines*) and reduced synchrony (*red open circle*) to sAM stimuli presented at 75 dB SPL and centered at 16 KHz. Gray line indicates mean spontaneous firing rate. Axes same as **(A)**.

Based on the decrease in markers of GABAergic inhibition seen at the anatomical level, GABA_A_ conductance was modified to reproduce the results seen at the level of the single neuron. Figure 10 shows an IC Sustained model with two DCN inputs and five DNLL high-pass inputs with LP rate and synchrony coding. Figure 10A (left) shows a voltage trace from the “young” model with full inhibition for an AM frequency of 16 Hz, demonstrating periodic firing at the modulation frequency. Upon reduction of GABA_A_ conductance by 50%, the neuron remained more depolarized (Figure 10A, right), and the synchronized firing was abolished (Figure 10B). The neuron remained LP rate tuned with higher firing rates (blue dashed line). This decrease in synchrony was similar to those observed at the single neuron level in the aged, as illustrated by responses of an aged neuron, A21, to sAM stimuli centered at 16 kHz, presented at 75 dB SPL (Figure 10C). This example neuron exhibited a low-pass rMTF with an rBMF of 8 Hz (rate – 30.4 spikes/s) and LP tMTF with a tBMF of 8 Hz (vs – 0.42).

**Figure 11:**
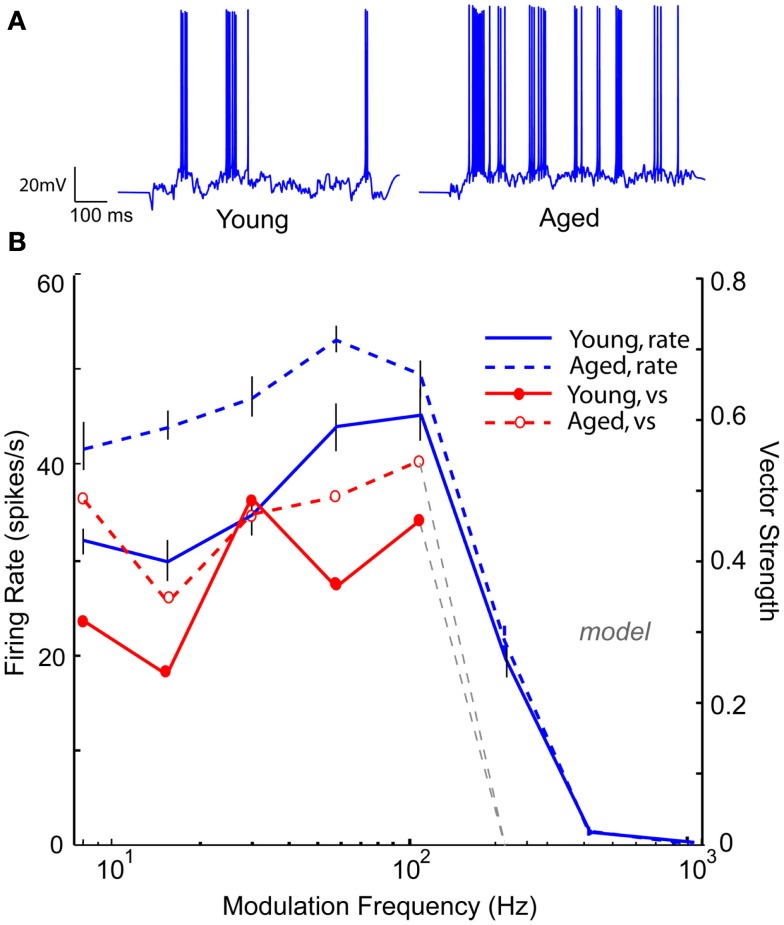
**Transformation of band-pass rMTF to low-pass rMTF by reducing GABA_A_**. IC Adapting model with 3 user-defined low-pass excitatory inputs (LSO sync tuning) and 3 VNLL band-pass inhibitory inputs (rBMF = 12 Hz). **(A)** Example voltage traces from individual trials at 16 Hz modulation frequency for a young (left) and aged (right) model neuron. **(B)** A band-pass rMTF (*solid blue*) from a model young neuron is transformed into a low-pass rMTF (*dashed, blue*) upon 50% reduction in GABA_A_. The original vector strength (*solid red*) decreased (*dashed, red*) but the cut-off frequency remained the same. Firing rate, in spikes/s, and vector strength are marked along opposing *Y*-axes and modulation frequency is marked along the *X*-axis in Hz.

Neuronal responses from aged animals in a previous study (Palombi et al., 2001) revealed a significant increase in LP rate tuned responses, whereas a slight increase was seen in aged animals in the present study. Figure 11 shows an IC Adapting model with four LSO LP excitatory inputs with six VNLL BP inhibitory inputs (rBMF = 12 Hz) with BP rate tuning. For 16 Hz AM, the voltage trace in the “young” model neuron (Figure 11A, left) shows a low but periodic firing rate. Upon decreasing GABA_A_ conductance by 50%, the “aged” model neuron transformed into a LP rate coded neuron, which can be seen in Figure 11B (blue dashed line) while maintaining or even exceeding the vs of the “young” model neuron (Figure 11B, red lines). In this example, decreasing inhibition allowed the synchronized excitatory inputs to reach threshold, generating a synchronized response with a higher rate than in the young (Figure 11A, right).

**Figure 12:**
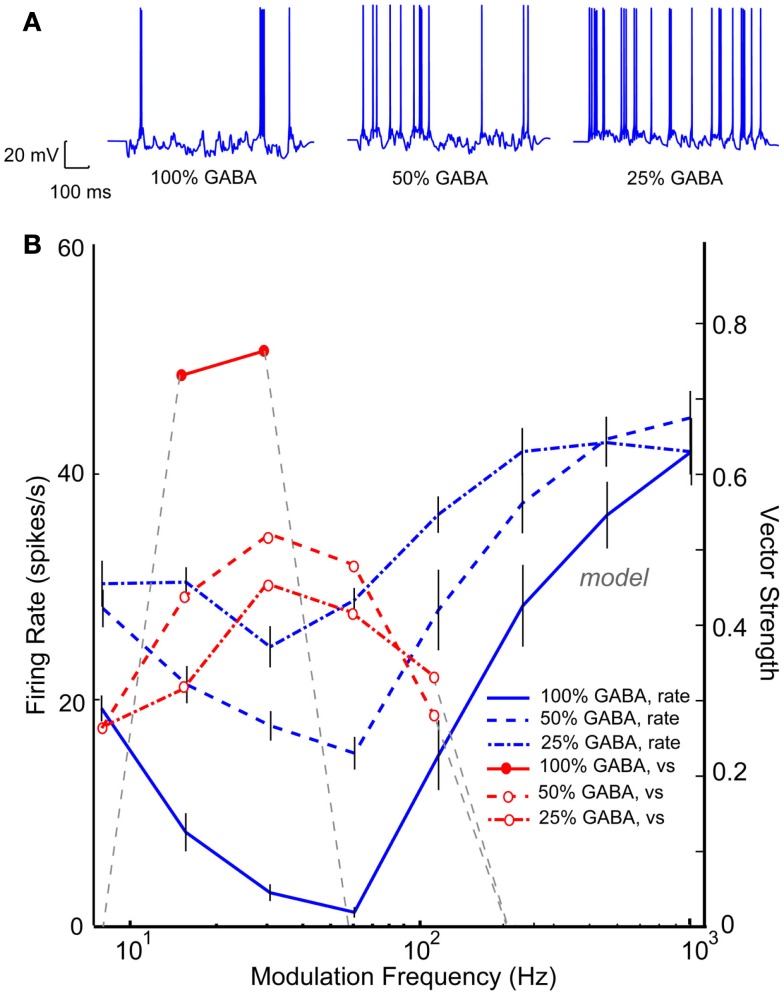
**Reduction in band-reject rMTFs due to reduction in GABA_A_**. IC Adapting model with 3 VCN inputs and 4 VNLL band-pass inputs (rBMF = 32 Hz). **(A)** Example voltage traces from individual trials at 16 Hz modulation frequency for a young (left) and aged (right) model neuron. **(B)** Changes in mean firing rate of a model neuron exhibiting band-reject rMTF (*solid blue*) with reduction in GABA_A_ to 50% (*dashed blue*) or to 25% (*dot-dashed, blue*). The original vector strength (*solid red*) reduced for both conditions (*dashed, red*, and *dot-dashed red* respectively). Firing rate, in spikes/s, and vector strength are marked along opposing *Y*-axes and modulation frequency is marked along the *X*-axis in Hz.

Band-reject neurons or neurons with a “worst modulation frequency” have been reported in a significant percentage IC AM responses in young animals (Krishna and Semple, 2000; Krebs et al., 2008). These neurons are thought to be the result of an excitatory AP input spike train that coincided with a tuned inhibitory input upon an IC neuron. An IC Adapting model using three VCN inputs and five VNLL BP inputs (rBMF = 32 Hz) produced a BR response with a minimum at 64 Hz and a synchronized range from 16 to 32 Hz, as seen in Figure 12B (blue and red solid lines). Upon reduction of this tuned inhibition by 50% (blue dashed lines), the rate at the worst modulation frequency increased. The neuron was still BR at 32–64 Hz, but the depth of rate modulation was much lower. Similar to Figure 11, reducing inhibition uncovered the synchrony of the excitatory inputs, resulting in synchronized responses from 8 to 128 Hz. Further reduction of 75% of the inhibitory conductance generated a high-pass rMTF (dash-dot lines), reflecting the VCN inputs and the lack of inhibition at high AM frequencies. This example demonstrates the complicated relationship between the balance in excitation and inhibition and the representation of temporal modulation. In this case, inhibition shaped selectivity for AM for both rate and synchrony in the “young” model, restricting the response to low modulation frequencies. Loss of inhibition resulted in a loss of selectivity but a more extensive range over which periodicity could be represented by a single IC neuron.

## Discussion

### Summary of results

In this study, neuronal responses were recorded in the IC of young and aged Fischer-344 rats to using sAM and nAM stimuli. In young animals, these responses were primarily classified into LP, BP, BR, and AP responses based on their rate tuning. Neuronal tMTFs also showed LP and BP characteristics. These major types of responses were recreated using a single compartment IC neuron model with voltage-gated ion channels and synaptic ligand-gated channel characteristics derived from previous *in vitro* and *in vivo* studies, including physiologically realistic input rates and synaptic depression.

Predictions based on changes in response patterns due to aging, primarily due to decreased inhibition, were motivated by changes in anatomical markers as well as changes in responses to the same AM stimuli from the IC of aged animals. In addition, it was demonstrated that changes in the balance between excitation and inhibition (Figure 7), as well as changes in recovery timing of synaptic depression (Figure 6) produced decrements in temporal processing consistent with data from aged animals. Hence this model provides a powerful framework for understanding the cellular mechanisms of temporal processing in the IC, and predicting how they may change in cases of aberrant processing, such as those in aging.

### Consideration of model parameters

The creation and use of the IC model uncovered important assumptions and needs for additional experimental data. A single compartment model of IC neurons was used in this study. Most neurons in the central nucleus of IC have disk-shaped morphology (Oliver, 1984; Peruzzi et al., 2000), where the dendrites are thick proximally and taper within about 50 μm, extending 100–150 μm from the cell body in the rat (Peruzzi et al., 2000). Many inhibitory synapses onto IC neurons are axosomatic (Ito et al., 2009), whereas excitatory synapses are found mainly along the dendrites (Oliver et al., 1999). Although dendritic filtering of synaptic inputs, which may be important in auditory brainstem neurons (Joris et al., 1998), was not accounted for, there are mechanisms in hippocampus and cortex that allow somatic EPSP amplitude to be largely independent of synaptic location (Magee and Cook, 2000). Hence a point model neuron was used as a first approximation lacking further evidence for how synapses and ion channels are distributed in IC neurons.

Although there are multiple sources of ascending excitatory and inhibitory projections to IC, studies of their synaptic properties, largely conducted using brain slices, have typically not dissociated the sources. Therefore, only the aggregate population synaptic characteristics are known (Smith, 1992; Wu et al., 2004; Sivaramakrishnan and Oliver, 2006), not the input-specific synaptic characteristics for each source. Moreover, the number of synaptic inputs used to model IC neurons has varied widely from 1 to 30 inputs (Reed and Blum, 1999; Shackleton et al., 2000; Nelson and Carney, 2004; Guerin et al., 2006). Data from *in vitro* recordings suggests a moderate number of inputs (1–10) converge on IC neurons (Smith, 1992; Wu et al., 2004). While this is clearly fewer than the number of synapses on a given neuron, it may provide an estimate of the number of functional levels of excitation or inhibition. Given that individual afferent axons often contribute more than one synapse onto a single neuron, it is useful to consider the functional levels that can be evoked by electrical stimulation as a lower estimate of the number of separate inputs evoked by sound. These numbers were sufficient to drive IC rates comparable to those *in vivo*. Some IC models have suggested the use of balanced inhibition, while others (Nelson and Carney, 2004) have suggested that inhibition should dominate. This was often the case in our model, where even in the presence of 4–6 inhibitory inputs, 2–3 excitatory inputs could drive model IC neurons to rates comparable to those *in vivo* (e.g., Figures 4, 5, and 10–12). Moreover, we have verified that BP IC responses can be generated by LP excitatory inputs with equal or higher corner frequencies than LP inhibitory inputs (Figures 4F,H), as suggested by Dicke et al. (2007). Another assumption in the model is that a given neuron receives only one type of excitatory or inhibitory input. There have been excellent studies demonstrating functional zones of inputs to the IC (Malmierca et al., 2005; Cant and Benson, 2006; Loftus et al., 2010), but whether different excitatory or different inhibitory inputs converge on individual IC neurons has not been established. Finally, the effects of local excitatory and inhibitory collaterals in IC (Herrera et al., 1989; Oliver et al., 1991; Paloff et al., 1992) have been omitted from this iteration of the IC model but would be likely to enhance or suppress the sustained responses of IC neurons, respectively.

### Comparison of the model with previous models

Experimental parameters for the present study were obtained from IC studies using brain slices (Peruzzi et al., 2000; Wu et al., 2004; Sivaramakrishnan and Oliver, 2006) and from *in vivo* intracellular recordings (Tan and Borst, 2007; Tan et al., 2007; Geis and Borst, 2009). From these studies, it was possible to obtain values for membrane properties, synaptic amplitudes and time courses, and short-term synaptic plasticity. The setup of input tuning curves to generate different IC outputs in our model overlapped with schemes in previous models (Cai et al., 1998a,b; Nelson and Carney, 2004; Guerin et al., 2006; Dicke et al., 2007), which in most cases focused on BP tuned IC neurons and used much faster synaptic time constants than have been measured for IC neurons. This does not diminish the functional insights gained from these models, but the current model can generate the spectrum of IC responses using more recent synaptic data and membrane properties. For example, *in vivo* it is known that synaptic depression and facilitation can have significant influence on information transmission (Elhilali et al., 2004; Wolfart et al., 2005), especially using more realistic patterns of activity (Hermann et al., 2009). Although spontaneous activity may affect the steady state magnitude of depression (Hermann et al., 2007), the spontaneous rate of IC inputs is often low (Tan et al., 2007, Figures 4, 5), and the spontaneous rates of many of the IC inputs are low (Yin and Chan, 1990; Bajo et al., 1999; Paolini and Clark, 1999), so it is still expected that some depression of inputs to the IC will be present for the initial sound-evoked activity. In addition, data from the calyx of Held synapses suggests that extracellular calcium concentration and intracellular calcium handling will affect the strength and time course of depression (Lorteije et al., 2009) and that *in vivo* extracellular calcium concentration is probably lower than typically used in brain slice experiments. However, activity dependent shifts in depression, or the balance of excitatory and inhibitory depression (Figure 6), may be an interesting way to regulate IC sustained activity. The advantage of using realistic intrinsic properties and synaptic properties for the IC is that they can be modified to make specific, testable mechanistic predictions about how changes in those properties, due to conditions such as aging or hearing loss, will affect IC model responses. In this study, the main focus has been on synaptic parameters that change, such as a decrease in GABAergic inhibition with age (Figures 10–12), but it is possible to test for other parameters that may be affected by aging, such as the influence of voltage-dependent ion channels, membrane properties, and changes in reversal potentials associated with impaired bioenergetics in aging (Mei et al., 1999; Melov, 2004).

### Comparison of *in vivo* responses with previous studies

The classification of rate MTFs followed earlier studies in the IC, with studies reporting LP, BP, and AP tuning (Rees and Moller, 1987; Langner and Schreiner, 1988; Rees and Palmer, 1989). Previous studies also reported BR rMTF tuning, as a worst modulation frequency (Krishna and Semple, 2000; Krebs et al., 2008). The BR tuning is thought to be due to the presence of tuned inhibitory inputs converging on an IC neuron along with an AP rate input, but it is also possible to get BR tuning with inhibition that precedes excitation (Figure A3 in Appendix). Many other neurons in this study showed a LP or BP tMTF, with the distributions of tBMFs and *F*_max_ similar to those reported in earlier studies in rats (Rees and Moller, 1987). Though temporal coding of periodic stimuli is not generated in the IC and is inherited from the lower auditory inputs, these responses may be sharpened due to the effect of inhibition mediated by GABA in young animals (Caspary et al., 2002; Zhang and Kelly, 2003). It is the reduction in inhibition estimated by a reduction in GAD 65/67 staining seen in this study (Figure 8), as well as reported previously in various other studies that is thought to be a contributing factor to altered temporal responses with age (reviewed in Caspary et al. (2008). The reduction in GABAergic inhibition has robust effects on rate coding and vs, with an overall increase in rate and more complex effects on vs. Rate changes can transform rMTFs from BP tuning to LP tuning or from BR to AP or high-pass (Figures 10–12).

### Mechanisms determining AM processing characteristics in young

The IC neuron model highlights several possible mechanisms of IC neurons that can influence responses to AM inputs, and it allows for adjustments of many parameters that modify intrinsic and synaptic properties.

Output response tuning to AM inputs can be inherited via combinations of different input tuning. Figure 3 shows responses to different types of afferent rate tuning, which are compared to the corresponding output responses. Coupled with AP inhibition, excitatory inputs exhibiting distinct rate characteristics often produce rate responses that inherit and display similar rate curves.

Not surprisingly, regions of few excitatory and numerous inhibitory inputs suppressed firing and either produced purely subthreshold responses, or responses with very low spike counts. More interestingly, this combination could transform AP VCN excitatory inputs to LP IC rMTF outputs (Figure 7C). Predictably, numerous excitatory and few inhibitory inputs produced large spike rates that are either non-synchronized or take on the characteristics of their inputs, such as the AP responses in Figures 7A,D. The best fits with data occurred when there were 1–3 more inhibitory inputs than excitatory inputs (Figures 4, 5), suggesting that the inhibition dominated models of Nelson and Carney (2004) may fit the data best.

The GABA_A_ IPSC decay time was another factor that significantly influenced the output response characteristics, particularly response rates. Increasing the IPSC decay value by 50% decreased spike rates at all modulation frequencies, due to larger hyperpolarization and increased summation of inhibitory inputs. This increase could also drive selectivity of the rMTF, as shown in Figures 7B,E, in a manner that was similar to increasing the peak conductance. Conversely, shortening IPSC decay values by 50% increased firing rates and reduced rMTF selectivity. This decrease sometimes reduced response vs measures and made the synchronized portion of its tMTF more similar to that of its inputs. Unlike IPSC decay time, adjustments to inhibition timing (±5 ms) relative to excitation produced only minor differences in rate or synchrony, similar to previous model studies (Nelson and Carney, 2004), which is probably due the integration of multiple inputs with multiple spikes per cycle. If the firing between the excitatory and inhibitory inputs were significantly correlated, rather than independently generated as they have been modeled in this study, delay would be more of a factor.

Finally, tuned rate responses could be obtained in a straightforward manner in the model using tuned excitatory inputs alone or in conjunction with tuned inhibitory inputs. This may be occurring for MSO inputs (Grothe, 1994) and for DNLL and VNLL inputs (Yang and Pollak, 1997; Zhang and Kelly, 2006). Tuned inhibition explains some results in young animals, such as a decrease in firing rate in the low AM frequency region of some BP units in IC generated by either BP or LP inhibitory inputs. However, it does not adequately explain on-rBMF inhibition and maintenance of BP tuning and rBMF during blockade of inhibition (Caspary et al., 2002). This appears to require additional mechanisms such as coincidence detection (Guerin et al., 2006) or cross-correlation (Nelson and Carney, 2004) except that slower synaptic time constants measured for IC neurons should be used. Further study is required to correlate subthreshold membrane potential contours with rMTF shape and with model responses, as was found in Geis and Borst (2009). However, Figures 7G–I from our study demonstrated that increasing the number of excitatory inputs smoothes the membrane potential contours, given a constant strength of inhibition. In addition, higher frequency inputs also smooth the membrane potential contours (Figure 7J), similar to Geis and Borst (2009).

### Manipulations in models that produce aging-like responses and comparison with other aged IC studies

Reduction in GABAergic inhibition was chosen for initial simulations of age-related changes in the IC model. Intrinsic parameters were largely ignored for this iteration of the model because there are very few data on age-related changes in membrane channels, though some changes have been observed with deafness or hearing loss (Kharkovets et al., 2000; Vale et al., 2003; Cui et al., 2007). A decrease in GAD65/67 has been observed in Figure 8 and a number of previous studies (Raza et al., 1994; Burianova et al., 2009), signifying that the amount of GABA available may be lower in aged animals. There are also indications of alterations of the GABA_A_ subunit composition (Backoff et al., 1999), suggesting that both pre- and post-synaptic components of fast inhibition in the IC are altered. A simple way to test the aggregate effect of these changes was to reduce the GABA_A_ conductance in the model, though a finer-scale modeling of amplitude and kinetics could be possible. Although Figure 8B suggests that presynaptic markers of excitatory terminals are not affected by aging, evoked glutamate release (Caspary et al., 1990a) and glutamate receptor subunit composition (Marianowski et al., 2000; Holt et al., 2005) can both be affected by aging, so simple changes in excitatory function can be tested in conjunction with decreased inhibition in the future. Finally, recovery from sound gaps (Walton et al., 1998) or sequential stimuli (Finlayson, 2002) suggested that recovery from depression may be affected by aging, and Figure 6 suggests that changing synaptic depression changes both rate and temporal selectivity in IC.

Manipulations that affected GABA_A_ conductance shaped tuning in ways consistent with previous studies of aged animals. For units with LP responses (Figure 10), rates increased and vector strength often decreased. For units whose BP tuning was shaped by LP or BP inhibition, reducing inhibition led to a shift to LP tuning (Figure 11). For units with BR tuning, created by LP or AP excitation and BP or preceding AP inhibition, reducing inhibition led to high-pass or AP rMTF responses (Figure 12). These effects are similar to those observed in IC studies of young rats (Caspary et al., 2002; Zhang and Kelly, 2003), where a subset of BP units reverted to LP responses during bicuculline administration. Many of the units still had BP tuning with elevated rates, suggesting that the BP tuning was inherited from inputs or generated by synaptic and cellular filtering in IC. The results are also consistent with our small sample of aged units and studies of AM in aged animals (Palombi et al., 2001), where there was a higher percentage of LP responses. These examples demonstrate the versatility of the IC model in testing and making predictions about consequences of different changes in IC synaptic and neuron properties, but were intended to be illustrative rather than exhaustive.

### Model predictions and gaps in necessary experimental data revealed by model

By using IC experimental data from rodents for model values where possible, construction of the model revealed experimental avenues for future study in order to more accurately simulate IC processing. The synaptic properties of the main ascending afferents to IC need to be dissociated and analyzed separately, for both the excitatory and inhibitory inputs. In addition, the anatomical convergence of afferents at the single neuron level needs to be examined in order to determine how many different ascending axons send terminals to a different neuron and whether different excitatory sources or different inhibitory sources often converge. Estimates were made based on current available data and the ability to recapitulate basic AM tuning with minimal manipulations, but this study has not focused on specific parameter fitting to individual neuron data. In addition, the inputs to the IC model were assumed to be normal, despite evidence that there are temporal processing deficits evident in single unit brainstem responses (Schatteman et al., 2008) and population neural responses that include brainstem and midbrain sources (Parthasarathy et al., 2010; Parthasarathy and Bartlett, 2011).

### Concluding remarks

**Figure 13:**
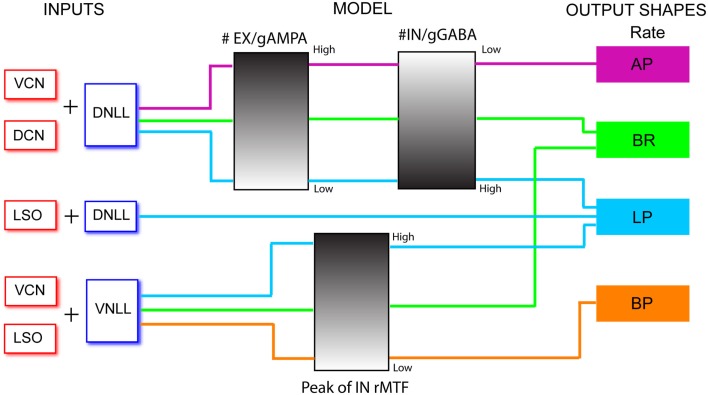
**Model output schematic**. Diagram describes possible rate output shapes generated by different combinations of inputs (*left*) and changes in model parameters (to be read left to right). Excitatory inputs are shown in red boxes and inhibitory inputs are shown in blue boxes. Adjustment of model parameters (*center*), such as number of excitatory or inhibitory inputs, magnitude of synaptic conductances of AMPA or GABA_A_, duration of inhibitory post synaptic currents (IPSCs), or frequency of peak inhibitory input rMTF can shape output rate response and lines through gradients indicate magnitude of each parameter. Similar colored lines correspond to different rate output shapes (*right*) produced by different combinations.

The IC model presented here, using realistic voltage-dependent membrane channels and synaptic ion channels, is able to produce all *in-vivo* rMTF responses described in this study (Figures 4, 5). Figure 13 shows a schematic of the IC model with the different ascending input combinations, key model parameters, and the resulting output rMTFs generated. LP inputs, such as the LSO inputs (Figure 2), preserved their rate shape if coupled with either AP or high-pass inhibition (DNLL). AP inputs (DCN, VCN) with a large number of inhibitory inputs or BP inhibition tuned to high (>64 Hz) frequencies also produced a LP rate response. AP inputs preserved their rate shape when they outnumbered inhibitory inputs or, similarly, had large AMPA conductances. There was an intermediate regime between LP and AP responses (Figures 7A–F) where AP inputs (VCN, DCN) coupled with DNLL inhibition produced BR responses. In addition, BR responses could be generated when the AP excitatory inputs were coupled with inhibition tuned to intermediate modulation frequencies. BP responses were occasionally generated for specific parameter combinations (Figures 7A–F), but they could be reliably generated from LP LSO inputs coupled with VNLL BP inhibition tuned to low (<32 Hz) frequencies or from LP inhibitory inputs with a lower LP corner frequency. Based on these observations, there were predictable changes in rMTF when one aspect of age-related change was simulated by reducing GABAergic inhibition. This could cause BP responses to become LP (Figure 11) or BR responses to become AP or high-pass (Figure 12). Taken together, it appears that IC outputs will reflect the rMTFs and basic tMTF characteristics of their inputs, but this simple relay is shaped by inhibitory inputs to transform the AM rate selectivity within the IC, which is greatly diminished by reduced inhibition such as that found in aging.

## Conflict of Interest Statement

The authors declare that the research was conducted in the absence of any commercial or financial relationships that could be construed as a potential conflict of interest.

## Figures and Tables

**Figure A1:**
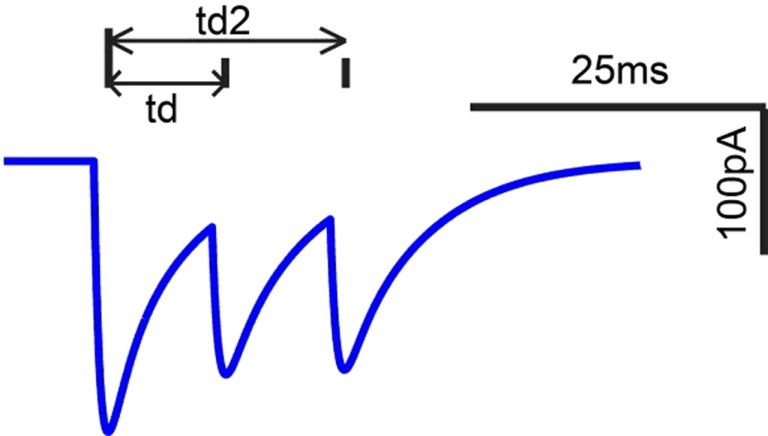
**AMPA current exhibiting synaptic depression**. *t*_d2_ and *t*_d_ refers to interval relative to current (third) synaptic event.

## Appendix

The ionic channels for the model are described by the following equation:

$$I_{\text{x}}=g_{\text{x}}\left(V_{\text{m}}-E_{\text{x}}\right)$$

Where *g*_x_ is the time-varying conductance for an individual ion channel multiplied by the difference between the membrane potential, *V*_m_ and the reversal potential for that ion channel, *E*_x_. The time course and magnitude of the activation and inactivation parameters are given for each of the channel equations listed below. The models were implemented in NEURON v7.1 (Hines and Carnevale, 2001) using a time step of 0.02 ms and a temperature of 34°C. Model cell capacitance for each type were set to a value of 1 μF/cm^2^. Times are in ms, voltages are in mV, concentrations are in mM, and currents (unless otherwise stated) are in mA/cm^2^.

### Fast, transient Na^+^ current (Traub et al., 2003)

$$\begin{array}{llll} & I_{\text{Na}}=g_{\text{Na}}m^{3}h\left(V_{\text{m}}-E_{\text{Na}}\right) & & \\ & \tau_{\text{m}}=1∕\left(\alpha_{\text{m}}+\beta_{\text{h}}\right) & & \\ & m_{\infty }=\alpha_{\text{m}}∕\left(\alpha_{\text{m}}+\beta_{\text{m}}\right) & & \\ & \alpha_{\text{m}}=\left[0.32\left(-\left(V_{\text{m}}+39\right)\right)\right]∕\left[e^{\left(-\left(V_{\text{m}}+39\right)∕4\right)}-1\right] & & \\ & \beta_{\text{m}}=[0.28*\left(\left(V_{\text{m}}+12\right)\right]∕\left[e^{\left(\left(V_{\text{m}}+12\right)∕5\right)}-1\right] & & \\ & \tau_{\text{h}}=1∕\left(\alpha_{\text{h}}+\beta_{\text{h}}\right) & & \\ & h_{\infty }=\alpha_{\text{h}}∕\left(\alpha_{\text{h}}+\beta_{\text{h}}\right) & & \\ & \alpha_{\text{h}}=0.128e^{\left(-\left(V_{\text{m}}+35\right)∕18\right)} & & \\ & \beta_{\text{h}}=4∕\left[1+\exp^{\left(-\left(V_{\text{m}}+12\right)∕5\right)}\right] & & \end{array}$$

### Potassium delayed rectifier current (Traub et al., 2003)

$$\begin{array}{llll} & I_{\text{Kdr}}=g_{\text{Kdr}}n^{4}\left(V_{\text{m}}-E_{\text{K}}\right) & & \\ & n_{\infty }=1∕\left[1+e^{\left(-\left(V_{\text{m}}+5.3\right)∕10.8\right)}\right] & & \\ & \tau_{\text{n}}=0.25+4.35e^{\left(\left(V_{\text{m}}+10\right)∕10\right)}\;\text{for}\;V_{\text{m}}<-10\text{mV} & & \\ & \tau_{\text{n}}=0.25+4.35e^{\left(\left(V_{\text{m}}+10\right)∕10\right)}\;\text{for}\;V_{\text{m}}>-10\text{mV} & & \end{array}$$

### Potassium current (TEA-sensitive; Sivaramakrishnan and Oliver, 2001; Traub et al., 2003)

$$\begin{array}{llll} & I_{\text{Ktea}}=g_{\text{Kdr}}n^{4}\left(V_{\text{m}}-E_{\text{K}}\right) & & \\ & n_{\infty }=1∕\left[1+e^{\left(-\left(V_{\text{m}}+7.2\right)∕8.9\right)}\right] & & \\ & \tau_{\text{n}}=0.25+4.35e^{\left(\left(V_{\text{m}}+10\right)∕10\right)}\;\text{for}\;V_{\text{m}}<-10\text{mV} & & \\ & \tau_{\text{n}}=0.25+4.35e^{\left(\left(V_{\text{m}}+10\right)∕10\right)}\;\text{for}\;V_{\text{m}}\geq -10\text{mV} & & \end{array}$$

### *I*_h_ channel (Koch and Grothe, 2003)

$$\begin{array}{llll} & I_{\text{h}}=g_{\text{h}}m\left(V_{\text{m}}-E_{\text{h}}\right) & & \\ & m_{\infty }=1∕\left[1+e^{\left(\left(V_{\text{m}}+79.5\right)∕9.8\right)}\right] & & \\ & \tau_{\text{m}}=1.475+1∕\left[e^{\left(-7.647-0.038{\text{V}}_{\text{m}}\right)+e\left(-1.533+0.046{\text{V}}_{\text{m}}\right)}\right] & & \end{array}$$

### I_h_ channel, onset (Koch and Grothe, 2003)

$$\begin{array}{l} I_{\text{h}}=g_{\text{h}}m\left(V_{\text{m}}-E_{\text{h}}\right)\\ m_{\infty }=1∕\left[1+e^{\left(\left(V_{\text{m}}+79.5\right)∕9.8\right)}\right]\\ \tau_{\text{m}}=3.21+1/\left[e^{\left(-7.63-0.042{\text{V}}_{\text{m}}\right)}+e^{\left(-1.22+0.052{\text{V}}_{\text{m}}\right)}\right] \end{array}$$

### Low-threshold Ca^2+^ current (*I*_T_; Huguenard and McCormick, 1992)

$$\begin{array}{llll} & I_{\text{T}}=P_{\text{CaT}}m^{2}hG\left(V_{\text{m}},Ca_{\text{o}},Ca_{\text{i}}\right) & & \\ & G\left(V,\text{C}{\text{a}}_{\text{o}},\text{C}{\text{a}}_{\text{i}}\right)=\left(Z^{2}F^{2}V_{\text{m}}/RT\right)\left[\left({\text{Ca}}_{\text{i}}-{\text{Ca}}_{\text{o}}e^{\left(-ZFV_{\text{m}}/RT\right)}\right)/\left(1-e^{\left(-ZFV_{\text{m}}/RT\right)}\right)\right] & & \\ & m_{\infty }=1∕\left(1+e^{\left(-\left(V_{\text{m}}+57\right)∕6.2\right)}\right) & & \\ & h_{\infty }=1∕\left(1+e^{\left(\left(V_{\text{m}}+81\right)∕4\right)}\right) & & \\ & \tau_{\text{m}}=0.612+1.0∕\left(\exp\left(-\left(v+132\right)∕16.7\right)+\exp\left(\left(v+16.8\right)∕18.2\right)\right) & & \\ & \tau_{\text{h}}=e^{\left(\left(V_{\text{m}}+467\right)∕66.6\right)}\;for\;V_{\text{m}}<-80 & & \\ & \tau_{\text{h}}=28+e^{\left(-\left(V_{\text{m}}+22\right)∕10.5\right)}\;for\;V_{\text{m}}\geq -80 & & \end{array}$$

### High-threshold Ca^2+^ current (*I*_L_; McCormick and Huguenard, 1992)

$$\begin{array}{llll} & I_{\text{L}}=P_{\text{CaL}}m^{2}hG\left(V_{\text{m}},Ca_{\text{o}},Ca_{\text{i}}\right) & & \\ & G\left(V,\text{C}{\text{a}}_{\text{o}},\text{C}{\text{a}}_{\text{i}}\right)=\left(Z^{2}F^{2}V_{\text{m}}/RT\right)\left[\left({\text{Ca}}_{\text{i}}-{\text{Ca}}_{\text{o}}e^{\left(-ZFV_{\text{m}}/RT\right)}\right)/\left(1-e^{\left(-ZFV_{\text{m}}/RT\right)}\right)\right] & & \\ & \alpha_{\text{m}}=1.6∕\left[1+e^{\left(-0.072\left(V_{\text{m}}-5.0\right)\right)}\right] & & \\ & \beta_{\text{m}}=0.02\left(V_{\text{m}}-1.31\right)∕\left[e^{\left(\left(V_{\text{m}}-1.31\right)∕5.36\right)}-1\right] & & \\ & m_{\infty }=\alpha_{\text{m}}∕\left(\alpha_{\text{m}}+\beta_{\text{m}}\right) & & \\ & \tau_{\text{m}}=1∕\left(\alpha_{\text{m}}+\beta_{\text{m}}\right) & & \end{array}$$

### Ca-dependent potassium channels (BK/SK; Aradi and Holmes, 1999; Sivaramakrishnan and Oliver, 2001)

$$\begin{array}{l} I_{\text{KCa}}=\left(g_{\text{bk}}+g_{\text{sk}}\right)\left(V_{\text{m}}-E_{\text{K}}\right)\\ g_{\text{bk}}=g_{\text{bk}}\text{bar}*rs^{2}\\ g_{\text{sk}}=g_{\text{sk}}\text{bar }q^{2}\\ q={\text{α}}_{\text{q}}/\left({\text{α}}_{\text{q}}+{\text{β}}_{\text{q}}\right)\\ r=7.5/\left(7.5+{\text{β}}_{\text{r}}\right)\\ s=1/\left(1+4/\left(1000*{\text{Ca}}_{i}\right)\right)\\ {\text{α}}_{\text{q}}\text{=0}{\text{.00246/e}}^{\left(\text{12log }10\left({\text{Ca}}_{i}\right)+28\right)/-4.5}\\ {\text{β}}_{\text{q}}\text{=0}{\text{.006/e}}^{\left(\text{12log }10\left({\text{Ca}}_{i}\right)+60.4\right)/35}\\ {\text{β}}_{\text{r}}\text{=0}{\text{.11/e}}^{\left(\left(V_{\text{m}}-35\right)/14.9\right)} \end{array}$$

### Low threshold potassium channel (Rothman and Manis, 2003; Zhou et al., 2005)

$$\begin{array}{llll} & I_{\text{KLT}}=g_{\text{KLT}}*w^{4}z*\left(V_{\text{m}}-E_{\text{K}}\right) & & \\ & W_{\infty }=1∕{\left(1+e^{\left(-\left(V_{\text{m}}+48\right)∕6\right)}\right)}^{1∕4} & & \\ & \tau_{\text{W}}=1.5+100∕\left(6e^{\left(\left(V_{\text{m}}+60\right)∕6\right)}+16e^{\left(-\left(V_{\text{m}}+60\right)∕45\right)}\right) & & \\ & Z_{\infty }=0.5+0.5∕\left(1+e^{\left(\left(V_{\text{m}}+71\right)∕10\right)}\right) & & \\ & \tau_{\text{z}}=50+1000∕\left(e^{\left(\left(V_{\text{m}}+60\right)∕20\right)}+e^{\left(-\left(V_{\text{m}}+60\right)∕8\right)}\right) & & \end{array}$$

### High threshold potassium channel (Rothman and Manis, 2003; Zhou et al., 2005)

$$\begin{array}{llll} & I_{\text{KHT}}=g_{\text{KHT}}*\text{(0}\text{.85}n^{\text{2}}\text{ + 0}\text{.15}p\text{)}*\text{(}V_{\text{m}}-E_{\text{K}}\text{)} & & \\ & n_{\infty }=1∕{\left(1+e^{\left(-\left(V_{\text{m}}+15\right)∕5\right)}\right)}^{2} & & \\ & \tau_{\text{n}}=0.7+100∕\left(11e^{\left(\left(V_{\text{m}}+60\right)∕24\right)}+21e^{\left(-\left(V_{\text{m}}+60\right)∕23\right)}\right) & & \\ & p_{\infty }=1∕\left(1+e^{\left(-\left(V_{\text{m}}+23\right)∕6\right)}\right) & & \\ & \tau_{p}=5+100∕\left(4e^{\left(\left(V_{\text{m}}+60\right)∕32\right)}+5e^{\left(-\left(V_{\text{m}}+60\right)∕22\right)}\right) & & \end{array}$$

### Rapidly inactivating potassium channel (Rothman and Manis, 2003)

$$\begin{array}{l} I_{\text{A}}=g_{\text{KA}}*(a^{4})b*c*(V_{\text{m}}-E_{\text{K}})\\ a_{\infty }={\left(1/\left(1+e^{\left(-\left(V_{\text{m}}+31\right)/6\right)}\right)\right)}^{1/4}\\ b_{\infty }={\left(1/\left(1+e^{\left(\left(V_{\text{m}}+66\right)/7\right)}\right)\right)}^{1/2}\\ c_{\infty }={\left(1/\left(1+e^{\left(\left(V_{\text{m}}+66\right)/7\right)}\right)\right)}^{1/2}\\ \tau_{a}=0.1+\left(100/\left(7e^{\left(\left(V_{\text{m}}+60\right)/14\right)}+29e^{\left(-\left(V_{\text{m}}+60\right)/24\right)}\right)\right)\\ \tau_{b}=1+\left(1000/\left(14e^{\left(\left(V_{\text{m}}+60\right)/27\right)}+29e^{\left(-\left(V_{\text{m}}+60\right)/24\right)}\right)\right)\\ \tau_{c}=10+\left(90/\left(1+e^{\left(-\left(66+V_{\text{m}}\right)/17\right)}\right)\right) \end{array}$$

### IC adapting model

The IC Adapting model is based on the Rebound-adapting response neurons found in IC, which are characterized by adaptation during sustained firing to depolarizing current and calcium rebound following hyperpolarizing currents (Sivaramakrishnan and Oliver, 2001; Koch and Grothe, 2003; Tan et al., 2007). The membrane potential of this neuron model is described by the following equation:

$$C*\left(dV∕dt\right)=I_{\text{Na}}+I_{\text{Kdr}}+I_{h}+I_{T}+I_{\text{L}}+I_{\text{KCa}}+I_{\text{syn}}+I_{\text{leak}}$$

Addition of the calcium activated potassium current *I*_KCa_ and the high threshold calcium current *I*_L_ contributes to the adaptation to depolarization. Inward rectification due to hyperpolarization is accomplished with the addition of *I*_h_. The low threshold calcium current *I*_T_ produces the depolarizing rebound following hyperpolarization.

The Na^+^ and delayed rectifier K^+^ current equations were taken from Traub et al. (2003). The maximal conductance values were adjusted to produce current values similar to those observed in Sivaramakrishnan and Oliver (2001; Table A1 in Appendix). Both the low-threshold and high-threshold calcium ion current equations were taken from a modeling study in the thalamus (Huguenard and McCormick, 1992). The values for maximal ion permeability *P*_Ca_ was adjusted to fit current values (Table A1 in Appendix). Sivaramakrishnan and Oliver (2001) observed potassium channels that were calcium-dependent and could be affected by either apamin (small conductance channels, SK) or charybdotoxin (large conductance channels, BK). We take existing kinetic models for SK and BK calcium-dependent potassium channels from a modeling study of hippocampal dentate granule cells (Aradi and Holmes, 1999) and adjusted the maximal conductance values *g*_bk_ bar and *g*_sk_ bar to fit current values found in Sivaramakrishnan and Oliver (2001; Table A1 in Appendix).

**Table A1:** **Model parameters for sustained, adapting, pause-build, and rebound-transient patterns**.

	Adapting	Sustained	Pause-build	Rebound-transient
Model surface area (μm^2^)	373.93	334.9	334.9	373.93
Na^+^ reversal potential (mV)	50	50	50	50
K^+^ reversal potential (mV)	−90	−90	−90	−90
Membrane leak reversal potential (mV)	−70	−70	−70	−70
Max. transient Na^+^ conductance (*g*_Na_; S/cm^2^)	0.2	0.1	0.1	0.2
Max. delayed rectifier K^+^ conductance (*g*_Kdr_; S/cm^2^)	0.1	0.1	0.1	0.1
Max. T-type Ca^2+^ permeability (*P*_CaT_)	0.00002 cm/s			0.00002 cm/s
Max. L-type Ca^2+^ permeability (*P*_CaL_)	0.00001 cm/s			0.00003 cm/s
Max. Ca^2+^ Activated K^+^ conductance (BK; S/cm^2^)	0.00226			0.0375
Max. Ca^2+^ Activated K^+^ conductance (SK)	0.03 S/cm^2^			0.005 S/cm^2^
Max. High-threshold K^+^ conductance (*g*_KHT_; S/cm^2^)		0.005		
Max. Low-threshold K^+^ conductance (*g*_KLT_; S/cm^2^)			0.0006	
Max. TEA-sensitive K^+^ conductance (S/cm^2^)		0.014	0.014	
Max. A-type K^+^ conductance (*g*_kA_; S/cm^2^)			0.00125	
Max. hyperpolarization-activated cation current (*g*_h_; S/cm^2^)	0.000218		0.0002	0.0001
Leak conductance	0.0000149	0.00019	0.000028	0.00005
AMPA reversal potential (mV)	0	0	0	0
NMDA reversal potential (mV)	20	20	20	20
GABA_A_ reversal potential (mV)	−80	−80	−80	−80

**Table A2:** **Onset model parameters**.

	Onset
Model surface area (μm^2^)	380.46
Na^+^ reversal potential	50 mV
K^+^ reversal potential	−85 mV
Membrane leak reversal potential	−73 mV
Max. transient Na^+^ conductance (*g*_Na_)	0.22 S/cm^2^
Max. delayed rectifier K^+^ conductance (*g*_Kdr_)	0.01592 S/cm^2^
Max. Low-threshold K^+^ conductance (*g*_KLT_)	0.0035 S/cm^2^
Max. TEA-sensitive K^+^ conductance	0.01592 S/cm^2^
Max. hyperpolarization-activated cation current (*g*_h_)	0.000577 S/cm^2^
Leak conductance	0.00025 S/cm^2^
AMPA reversal potential	0 mV
NMDA reversal potential	20 mV

**Table A3:** **Synaptic current parameter values**.

	A	tau1 (ms)	tau 2 (ms)
AMPA	1.0526	0.5464	6
NMDA	0.56	32	50
GABAA	1.4085	3	15

**Table A4:** **AMPA and GABA depression equation constants**.

	AMPA	GABA
A1	0.378	1
A2	0.622	–
A3	115.4	–
A4	115.3	–
TauR (ms)	63.73	16.85
TauR2 (ms)	3.32	–
TauR3 (ms)	69.7	–
TauR4 (ms)	70.46	–

**Table A5:** **Figure parameters**.

Figure	Model type	Ex	IN	AMPA (nS)	NMDA (nS)	GABA_A_ (nS)	GABA decay (ms)	(mV)
Figure 3	A	3 LSO	2 DNLL (AP)	5	1.5	3	15	−60
Figure 4, low-pass	A	2 VCN	4 DNLL (HP)	5	1.5	4	15	−56
Figure 4, band-pass	S	3 LSO*	6 DNLL* (LP)	5	1.5	3	12	−60
Figure 5, all-Pass	A	3 DCN	2 DNLL (AP)	5	1.5	3	20	−56
Figure 5, band reject	A	2 VCN	6 VNLL (36 Hz)	5	1.5	4	15	−60
Figures 6A,B	A	3 DCN	2 DNLL (AP)	5	1.5	3	15	−56
Figures 6C,D	A	2 VCN	4 DNLL (HP)	5	1.5	3	15	−56
Figure 10	S	2 DCN	5 DNLL (HP)	5	1.5	3, 1.5	15	−56
Figure 11	A	4 LSO	6 VNLL (12 Hz)	5	1.5	4, 2	15	−60
Figure 12	A	3 VCN	5 VNLL (32 Hz)	5	1.5	4, 2, 1	15	−56

**User-defined low-pass inputs were used for Figures 4F,H. A, adapting; S, sustained*.

### IC sustained model

The sustained model is based on the sustained-regular cells, which have a nonadapting sustained firing pattern in response to depolarizing current.

$$C*\left(dV∕dt\right)=I_{\text{Na}}+I_{\text{Kdr}}+I_{\text{Ktea}}+I_{\text{KHT}}+I_{\text{syn}}+I_{\text{leak}}$$

The Na^+^ and delayed rectifier K^+^ current equations were taken from Traub et al. (2003). The maximal conductance values were adjusted to produce current values similar to those observed in Sivaramakrishnan and Oliver (2001; Table A1 in Appendix). Sivaramakrishnan and Oliver (2001) identified tetraethylammonium chloride (TEA-Cl) and 4-aminopyridine (4-AP) sensitive delayed rectifier K^+^channels. For the Sustained, Pause-Build, and Onset models, we have added a TEA-sensitive delayed rectifier channel, *I*_Ktea_ by adjusting the steady-state activation equations from the delayed rectifier current *I*_Kdr_. Equations for a high threshold potassium channel *I*_kHT_ were taken from a modeling study in medial superior olive (Zhou et al., 2005). Conductance values for *I*_KHT_ were adjusted to fit firing patterns to injected current pulses.

### IC pause-build, rebound-sustained, rebound-transient, onset models

The pause-build model was created by modifying the IC Sustained Model and adjusting channel conductance values as shown in Table A1 in Appendix, following the findings in Sivaramakrishnan and Oliver (2001). An A-type potassium conductance was added, along with a small *I*_h_ current, while the high-threshold potassium conductance was removed and the leak conductance were lowered somewhat.

The Rebound-Sustained model was created by modifying the IC Sustained Model, adding in a T-type calcium permeability (0.00002 S/cm^2^) and a weak *I*_h_ current (0.0000644 S/cm^2^).

The Rebound-Transient model was created by modifying the IC Adapting Model and adjusting the BK and SK Ca^2+^ channel conductance values such that the BK conductance was larger than the SK conductance, as reported by Sivaramakrishnan and Oliver (2001).

The Onset model, like the Sustained and Pause-Build model, has 4-AP and TEA-sensitive components of the delayed rectifier K+ current (Table A2). In addition, a low-threshold K+ current modeled from VCN neurons (Rothman and Manis, 2003) is also added. The kinetic equations for activation were adjusted according to Koch and Grothe (2003) to reflect rapid activation of *I*_h_ in IC onset neurons. The conductances of each were adjusted to fit firing patterns found in Sivaramakrishnan and Oliver (2001).

### Generation of input spike times

Given the rMTF and tMTF of the input sources (Figure 2), the average spikes per cycle were generated. A standard deviation of 50% of the average spikes per cycle spikes was typically used, constrained by the total number of spikes per trial. Spike times were generated by selecting from a Gaussian distribution whose width was chosen to generate the vector strength given by the tMTF of the input. The Gaussian widths were determined empirically. A refractory period of 1.5 ms was used, encompassing absolute and relative refractory periods. This produced a series of spike times whose characteristics matched the input rates and vector strengths.

For LSO and VCN input sources, spike trains were generated such that the stimulus onset (∼15 ms) had a higher average spikes per cycle than the sustained portion of the input train (∼735 ms). The onset to sustained average spikes per cycle was set to a ratio of 4:1; however, the overall average spikes per cycle remained the same. This adjustment was made to match PSTH spike data taken from studies in LSO and VCN (Adam et al., 1999; Paolini and Clark, 1999).

### Generation of synaptic currents

The synaptic current to the computational model is given by the following equation:

$$I_{\text{syn}}=\sum I_{\text{EX}}+I_{\text{IN}}$$

where *I*_EX_ and *I*_IN_ are the sum of all independent excitatory or inhibitory synaptic inputs, respectively. The general equation for synaptic current is given as

$${\text{I}}_{\text{syn(}i\text{)}}\text{ = }g\text{(}V\text{,}t\text{)}*\text{(}V-E_{\text{rev}}\text{)}$$

where the term *g* is voltage and time dependent and can be described in the following equation:

$$g\text{(}V\text{,}t\text{) = }g_{\text{max}}*p*\text{[A}*\text{(exp(}-t\text{/tau1)}-\text{exp}(-t\text{/tau2))]}$$

Values for A, tau1 and tau2 are given in Table A3 for the AMPA, NMDA, and GABA_A_ currents.

The term *p* refers to a scaling factor that reduces the synaptic conductance due to short-term synaptic plasticity. This scaling factor is dependent on the timing of the two preceding synaptic events and modeled from voltage clamp data from Wu et al., 2004. The general equation describing scaling due to short-term synaptic plasticity is given as follows:

$$\begin{array}{ll} P_{\text{AMPA}}=(1-\text{A1}*\text{exp(}-t_{\text{d}}\text{/tau}R\text{)}-\text{A2}*\text{exp(}-t_{\text{d}}\text{/tau}R\text{2))}\\ & \;*\text{(1 + A3}*\text{exp(}-\;t_{\text{d2}}\text{/tau}R\text{3)}\;-\;\text{A4}*\text{exp(}-\;t_{\text{d2}}\text{/tau}R\text{4))}\\ {\text{P}}_{\text{GABAA}}=(1-\text{A1}\;*\;\text{exp(}-t_{\text{d}}\text{/tau}R\text{))} \end{array}$$

Here *t*_d_ refers to the time elapsed from the previous synaptic event in the given synaptic input. The value of *t*_d2_ refers to the time elapsed from the second previous synaptic event in the given synaptic input. A schematic depicting *t_d_* and *t_d2_* for a series of evoked AMPA currents is shown in Figure A1. If only two synaptic events are present, the values of A3 and A4 are zero. The equation is an approximation to the time course of synaptic depression, with values of A1–4 and TauR1–4 chosen in order to best fit the EPSC amplitude ratios described in Wu et al. (2004).

For NMDA receptor dependent excitatory currents, *g* is also voltage-dependent and given by the following equation:

$$B\text{ = 1/(1 + 0}\text{.28}*\text{exp(}-\text{0}\text{.062}*V_{\text{m}}\text{))}$$

### Generation of band-pass and band-reject responses

Caspary et al. (2002) found band-pass tuning in the presence of bicuculline, suggesting inhibition may not be necessary for band-pass rate tuning. We recreate band-pass responses shown above using an Adapting model and three excitatory VCN inputs with onset EPSPs exhibiting adaptation (Figure A2 in Appendix). We use inputs using low input (50 spikes/s) rates, reduced AMPA and NMDA conductances (50 and 20%, respectively), and a more hyperpolarized membrane potential (−64 mV).

Band-reject responses were created using 3 VCN excitatory and 3 DNLL all-pass inhibitory inputs. We varied the input timing such that the inhibitory input either preceded (Figure A3 in Appendix) or lagged excitation (not shown). When inhibition was coincident with (Figure A3 in Appendix, green line) or briefly preceded excitation (Figure A3 in Appendix, 0, −1, −3 ms relative to excitation), band-reject responses were observed. If inhibition preceded excitation by >3 ms, all-pass responses were observed (Figure A3 in Appendix, red dashed line).
